# PPLook: an automated data mining tool for protein-protein interaction

**DOI:** 10.1186/1471-2105-11-326

**Published:** 2010-06-16

**Authors:** Shao-Wu Zhang, Yao-Jun Li, Li Xia, Quan Pan

**Affiliations:** 1 Institute of Control and Information, School of Automation, Northwestern Polytechnical University, Xi'an, China; 2 Molecular and Computational Biology Program, Department of Biological Sciences, University of Southern California, Los Angeles, USA

## Abstract

**Background:**

Extracting and visualizing of protein-protein interaction (PPI) from text literatures are a meaningful topic in protein science. It assists the identification of interactions among proteins. There is a lack of tools to extract PPI, visualize and classify the results.

**Results:**

We developed a PPI search system, termed PPLook, which automatically extracts and visualizes protein-protein interaction (PPI) from text. Given a query protein name, PPLook can search a dataset for other proteins interacting with it by using a keywords dictionary pattern-matching algorithm, and display the topological parameters, such as the number of nodes, edges, and connected components. The visualization component of PPLook enables us to view the interaction relationship among the proteins in a three-dimensional space based on the OpenGL graphics interface technology. PPLook can also provide the functions of selecting protein semantic class, counting the number of semantic class proteins which interact with query protein, counting the literature number of articles appearing the interaction relationship about the query protein. Moreover, PPLook provides heterogeneous search and a user-friendly graphical interface.

**Conclusions:**

PPLook is an effective tool for biologists and biosystem developers who need to access PPI information from the literature. PPLook is freely available for non-commercial users at http://meta.usc.edu/softs/PPLook.

## Background

Protein-protein interactions (PPI) execute many critical biological activities, including signal transduction and metabolic activity. Understanding these interactions can help in disease diagnosis, prevention and treatment. Although many efforts have been made to create databases that store the verified PPI information in a structured form, much PPI interaction information still remains unmined as unstructured text. While biomedical literature databases, such as MEDLINE databases http://www.ncbi.nlm.nih.gov contain such information, the structural features that would favor automatic access and data processing by computer are lacking. It goes without saying that manual extraction is both error-prone and time consuming. Moreover, it becomes potentially impossible to manually extract PPI information in a high throughput manner. Therefore, the quick and easy extraction and visualization of PPI relationships from biomedical text is an attractive research goal.

Thus far, more than 19 million citations of articles, journals, books and technical reports are available in the MEDLINE database. Many other databases, such as the DIP [1], MINT [2], IntAct [3], BioGRID [4], have been built by manually annotation to store the processed PPI data. However, a lot of PPI information still hides in the biomedical text literatures. Because a formal structure narrating the natural language of these documents is lacking, the task of mining and retrieval of PPI information is quite complex [5].

Several international systems now exist which can analyze literature databases (e.g., MEDLINE) to provide users with a summary of relevant biological information services [6-12], such as PIE [12] and iHOP [11], but both systems have met with only limited success. PIE extracts PPI from text-driven search results derived from papers or keywords, and iHOP lists related key sentences with a given query, but both do not visualize and classify the results. In addition, Suiseki[8] and BioBiblioMetrics [6] both focus on the extraction and visualization of protein-protein interaction, but without any classification and statistics. GENIES [7] retrieves molecular pathways from journal articles, and the MedScan [9] system uses whole sentence analysis technology to extract PPI from the MEDLINE database, but it does not support classification and offers no statistical function for the search results. Furthermore, its use requires commercial licensure. In 2005, Cooper and Kershenbaum applied a combination of approaches, including linguistics, statistics and information graphics, to discover protein-protein interaction [13] but they have not yet developed software for the system.

In this paper, we introduce a new PPI extraction tool, PPLook, which is based on the OpenGL graphics interface technology and uses a keywords dictionary pattern matching approach [14] as its core natural language processing (NLP) algorithm. Pattern matching is one of the PPIs extracting methods, and parsing approach is another method [15]. Given a query protein name, PPLook can search for the interacting proteins and show the results in a three-dimensional display. In addition, PPLook has incorporated many useful search functions, including heterogeneous search, classification, statistics, and resource integration.

## Implementation

**Figure 1:**
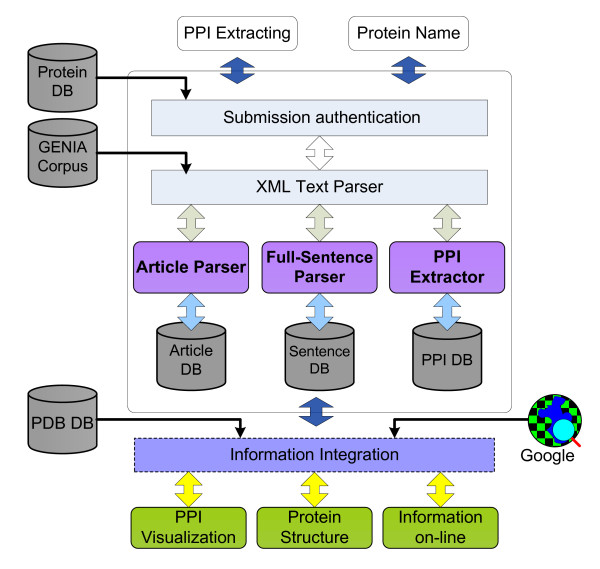
**Architecture of PPLook**. PPLook contains four modules: (i) submission authentication module aims to identify whether the query word is a protein name or not; (ii) article parser module acts as a collector to pick up sentences containing the query protein name; (iii) full-sentence parser module determines the PPI and (iv) PPI visualization module displays the PPI in the form of a 3-D graph.

PPLook is a four-tier information extraction system based on a full-sentence parsing approach. Conceptually, it contains four modules: (i) submission authentication which aims to identify whether the query word is a protein name or not; (ii) an article parser which acts as a collector to pick up sentences containing the query protein name; (iii) a full-sentence parser which determines the PPI by keywords dictionary pattern-matching and (iv) PPI visualization which displays the PPI in the form of a 3-D graph. Figure 1 shows the architecture of PPLook.

### Corpus

Annotated corpora are important to the development and evaluation of protein-protein extraction systems, and there are several available annotated corpora. As our simulation dataset, we chose the GENIA V3.02 Corpus [16], which can be downloaded freely from the following website: http://www-tsujii.is.s.u-tokyo.ac.jp/GENIA/home/wiki.cgi?page=GENIA+corpus.

### Submission authentication

Before extracting PPI information, we need to verify whether the input words represent a legitimate protein name. The protein name authentication module uses the GENIA Tagger tool to verify the input words [17]. A two-way reasoning algorithm was adopted to execute the tagging of protein names [18]. First, the two-way algorithm trains relevant parameters using the Penn Treebank corpus [19] as the training set, and then it analyzes the user's keywords to determine if the words are a protein name or not. The precision of the system of identifying the protein name for GENIA corpus is 98.26% http://www-tsujii.is.s.u-tokyo.ac.jp/GENIA/home/wiki.cgi?page=GENIA+corpus.

### XML text parser

GENIA Corpus is an XML-tagged text, which has been structured and annotated. Based on rules of the XML editor, an XML text parser was developed to read out the protein names and protein catalog.

### Article parser

Parsing occurs in two steps. In the first step, the article parser checks whether the dataset contains the given protein. In this procedure, the GENIA Tagger tool [17] is used to find all proteins contained in the dataset using a part-of-speech tagger. Then, by comparing the given protein with proteins contained in the dataset, the article parser module picks up all the sentences that contain the query protein. Because PPI information may be available from these sentences, PPLook scans the keywords (listed in Table 1) in all of these sentences and saves the sentences that contain one or more keywords.

**Table 1:** Statistical results of six key words.

ID	Key words	Frequencies	Recall (%)	Precision (%)
1	interact	538	89.1	96.1
2	bind	415	80.9	90.2
3	complex	1625	86.6	95.3
4	regulate	617	86.4	92.7
5	activate	1613	82.8	91.3
6	associate	483	80.4	92.5

### Full-Sentence parser and PPI Extractor

In the second step, the full-sentence parser identifies PPI from documents classified by the article parser. In order to find more and exact PPI patterns, we carried out statistical analysis of word frequency with our developed statistical tool of word frequency based on the statistical principles related to the verb.

Using the rules of pattern matching and part-of-speech [14], we tested GENIA V3.02 Corpus to find common ways of describing interactions, and examined approximately 30 different verbs (e.g. 'activate', 'inhibit', 'modulate', 'suppress', 'isolate', 'promote', 'characterize'). Considering the recall/precision of the keywords appeared in GENIA V3.02 Corpus, the six keywords (interact, associate, bind, complex, activate and regulate) and their corresponding patterns were selected to define the relationship between proteins. The recall/precision of six keywords and their corresponding PPI patterns are listed on Table 1 and Table 2 respectively.

**Table 2:** Keywords and corresponding PPI patterns

Keywords	Patterns
Interact	A interact with B
	Interaction of A (with | and) B
	Interaction(between | among)
	A - B interact
	A and B interact
Associate	A associate with B
	Association between A and B
	Association of A (with | and) B
	A and B associated with each other
Bind	Binding of A to B
	A and B bind
	Binding between A and B
	A bind B
Complex	A (- |/) B complex
	A and B complex
	complex A and B
	A complex with B
	Complex...contains B...
	A complex B
Activate	A activate B
Regulate	A regulate B

### Classification and statistic function

According to the taxonomy of GENIA Ontology, some entities (proteins) involved in reactions were classified semantically for the GENIA corpus. We adopted this semantic classification to the PPLook system. After the users select the protein semantic class which the users want to search interacting with a query protein, the PPLook can give the count number of how many this semantic class proteins interact with the query protein. In addition, the PPLook can also give the literature count number of the articles appearing the interacting relationship about the query protein. The literature count number supports the reliability of search results. If the literature count number is bigger, the confidence of this PPI is higher.

### PPI visualization and resources integration

PPLook system employs OpenGL technology for PPI visualization. The OpenGL graphics system is hardware for the GL graphics library software interface [20,21]. It provides powerful functions to create complex three-dimensional objects, such as balls, rings, cylinders, and polyhedrons. In PPLook, the extracted PPIs are well distributed to a sphere with the OpenGL. Each ball represents a protein. Different semantic classes of protein are represented by different colors. The stick-like connection between two proteins indicates their interaction relationship. Based on the inter-library web service search framework [22], PPLook provides a heterogeneous search engines [23] that can link to the PDB search engine and Google search engine, thus letting users directly proceed to online search to gather related information about their proteins of interest.

## Results and Discussion

**Figure 2:**
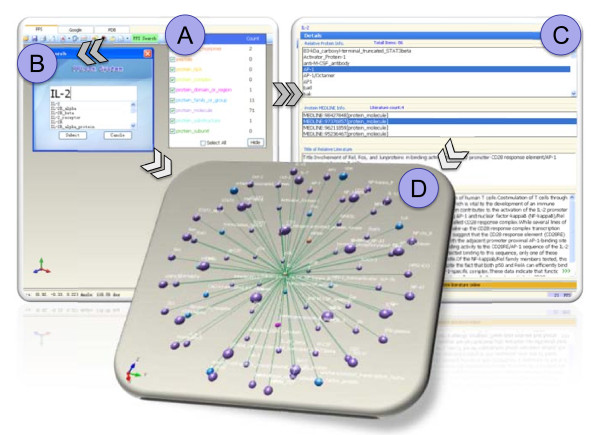
**An example of PPLook search results for the protein IL-2**. (A) is the protein semantic class selection window, (B) is the IL-2 protein input window, (C) is the text results output window, and (D) is the 3-D display output window.

Figure 2 shows an example of protein IL-2. (A), (B), (C) and (D) indicate protein semantic class selection, IL-2 protein input, text results, output window and 3-D display output window, respectively. The user just input a protein name and press the enter key. PPLook then provides an abundance of related information, such as PPIs, the protein structure, article number related to the query protein, and statistical counts of semantic class protein.

### 3D PPI viewer

Using OpenGL programming, PPI results returned are displayed in 3-D style. The user can either zoom in/out or rotate the PPI network in the main window. The user can also change the size and the color of balls or the length and color of lines according to their needs.

### Circular extraction

Assume that A is the first query protein. In this case, PPLook provides a circular extraction function that can help users extract PPI information of another protein B which interacts with protein A based on the outlink to B within the first query. The user just needs to click on protein B listed on the right side output window. The PPI information of protein B will immediately return on the main window.

### Heterogeneous search engines

Normally, users not only want to know PPI information of interest proteins, but also related information about the query protein, such as structures and published articles.

**Figure 3:**
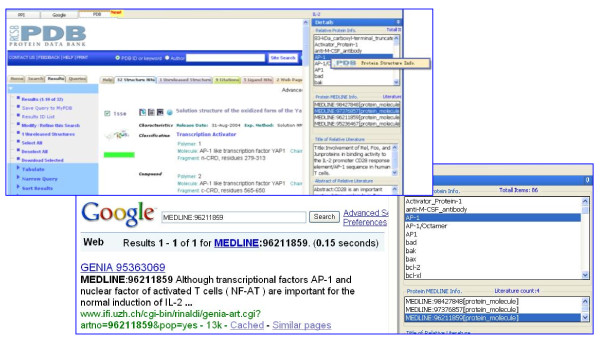
**Heterogeneous Search results: Structure information and MEDLINE results for the protein IL-2**. The top left window is the protein IL-2 structure information coming from PDB database. The bottom right window is the Google search results.

In the PPLook system, we developed heterogeneous search engines which include PPI search engine, PDB search engine and Google search engine. Users can get a unique answer that satisfies conjunctive queries where each query can be routed to a specialized engine. By heterogeneous search engineer, PPLook enables cross-references when users require PPI information, protein structures and published articles simultaneously. Otherwise, users have to use several specialized search engines to get what they want. Figure 3 shows structural data and Google search results for the protein IL-2.

### Data table output

PPLook provides many kinds of output data in a format that meets users' needs. The interaction protein names and corresponding literature appeared in the results can be exported as text files. The 3-D PPI network can also be saved in a design format (PPLook format) or in bitmap format. All PPI information can be printed whenever necessary.

### Performance of extracting PPI information with PPLook

The recall, precision and F value for assessment of the PPLook tool are respectively defined as:(1)(2)(3)

Where *TP *is the number of PPI extracted correctly by PPLook, *TP*+*FN *is the number of PPI in the dataset, *TP*+*FP *is the number of PPI retrieved by PPLook.

To evaluate the reliability of PPLook tool, we used the following search query on the PubMed web interface: "Humans" [MeSH] AND "Blood Cells" [MeSH] AND "Transcription Factors" [MeSH] to retrieve the corresponding records from MEDLINE database, then constructed a test dataset which concludes 415 abstracts. Based on interacting domain, gene compression profile and gene ontology (GO) annotation, we annotated the test dataset and identified the PPI by hand, and the annotated dataset which concludes 116 abstracts is shown in Additional file 1. Then, the test dataset was converted to XML format file with XML constructor, and also inputted to the PPLook tool. The average recall/precision and F of PPI extraction are 85.6%, 73.9% and 0.897 respectively. The average time of searching PPI of a query protein is about 1.2 s (100 times search randomly). The results show that PPLook is a valuable automated data mining tool of PPI from text literatures.

## Conclusions

In this paper, we introduced a useful tool, PPLook, which uses an improved keywords dictionary pattern-matching algorithm to extract protein-protein interaction information from biomedical literature. Based on the OpenGL graphics interface technology, visual methods were adopted to show the results of protein-protein interaction in three dimensional stereoscopic displays. PPLook can also provide users with more interactive features, such as the count of the semantic class protein and the number of articles appearing PPI information of query protein, the MEDLINE access number, title and abstract of related literature, as well as integrated resources and heterogeneous search functions.

Prospectively, PPLook will add more functions that include extracting PPI information for users submitting text articles or constructing complex PPI networks, both directed and undirected. In addition, based on the new MEDELINE database, we will develop a more complete XML-tagged text corpus in order to enhance the robustness of PPLook's search results.

## Availability and requirements

PPLook is freely available for non-commercial users at http://meta.usc.edu/softs/PPLook.

Operating system(s): Windows (2000, XP) 32-bit, 64-bit, requires .NET Framework version 2.0.

Hardware requirements/recommendations: Pentium III or later for Windows, Color display capable of 1024 X 768 pixel resolution.

Programming language: C++

## Authors' contributions

SWZ developed and implemented the tool and drafted the manuscript. YJL designed the software module, implemented and tested the codes. LX and QP participated in design and implementation. All authors read and approved the final manuscript.

## Supplementary Material

Additional file 1 **An annotated PPI dataset**. Click here for file
