# Supplementary material for: PPLook: an automated data mining tool for protein-protein interaction
Source: BMC Bioinformatics. 2010 Jun 16;11:326. doi: 10.1186/1471-2105-11-326 (PMC2906489; doi:10.1186/1471-2105-11-326)
Supplement: Additional file 1 — An annotated PPI dataset. [file 1471-2105-11-326-S1.DOC]

Annotated Protein-Protein Interaction (PPI) Dataset

MEDLINE:93194904

**Cell type- and stage-specific expression of the CD20/B1 antigen correlates with the activity of a diverged octamer DNA motif present in its promoter.**

The CD20(B1) encodes a B cell-specific protein involved in the regulation of human B cell proliferation and differentiation. Studies with 5' deletion CD20 promoter-CAT constructs have previously revealed two regions of the promoter between bases -186 and -280 and between bases -280 and -454 which contained positive regulatory elements. In this study we identified a sequence element present in the most proximal region located between bases -214 and -201, TTCTTCTAATTAA, which is important in the high constitutive expression of CD20 in mature B cells and the induction of CD20 in pre-B cells. This sequence element was referred to as the BAT box and its deletion significantly reduced the activity of a CD20 promoter-CAT construct in B cells. Mobility shift assays with various mutant probes and B cell nuclear extracts demonstrated that the core sequence TAAT was essential for binding to this site. Cross competition experiments with an octamer sequence from the Ig heavy chain promoter, the BAT box, and a TA-rich sequence present in the CD21 promoter revealed that all three sequences bound the same nuclear proteins suggesting that the BAT box binding proteins were Oct-1 and Oct-2. Southwestern blotting and UV cross-linking studies confirmed that the BAT box binding proteins were Oct-1 and Oct-2. The affinity of the BAT box binding proteins for the BAT box was approximately 25-fold less than for the octamer sequence and the BAT box binding proteins dissociated from the BAT box 10-fold more rapidly than from the octamer sequence. Despite this lower affinity, a trimer of the BAT box sequence was as efficiently transactivated by an Oct-2 expression vector as was a trimer of the octamer sequence in HeLa cells. The BAT box and Oct-2 were also implicated in the induction of CD20 in the pre-B cell line, PB-697, via phorbol esters. The induction of CD20 mRNA was temporally associated with induction of Oct-2 mRNA and a BAT box-deleted CD20-CAT construct, in contrast to the wild type, was poorly induced by phorbol esters. Together these results suggest that the BAT box binding proteins are important in the B cell specific expression of CD20 and perhaps CD21.

MEDLINE:93154317

**The Epstein-Barr virus nuclear antigen 2 interacts with an EBNA2 responsive cis-element of the terminal protein 1 gene promoter.**

The Epstein-Barr virus protein EBNA2 acts as a transcriptional activator of cellular and viral genes and plays a crucial role in the immortalization of human primary B-cells by EBV. We have shown previously that EBNA2 transactivates the promoters of the latent membrane antigens LMP, TP1 and TP2. The promoter of the TP1 gene was chosen as a model system to study the molecular mechanism of EBNA2 mediated transactivation. To identify an EBNA2 dependent cis-acting element, various TP1 promoter-reporter gene constructs were transfected in the absence and presence of an EBNA2 expression vector into the established B-cell line BL41-P3HR1. We were able to delineate an 81 bp EBNA2 responsive region between -258 and -177 relative to the TP1 RNA start site. The element worked in either orientation and could mediate EBNA2 dependent transactivation on a heterologous promoter. Electrophoretic mobility shift assays revealed three specific protein-DNA complexes formed with sequences of the EBNA2 responsive element. Two of these were not cell type specific, but the third was detected only in EBNA2 positive cell extracts. Gel-shift analysis in the presence of EBNA2 specific monoclonal antibodies revealed that EBNA2 is a component of the third complex. Thus, these experiments demonstrate that EBNA2 interacts with an EBNA2 responsive cis-element of the TP1 promoter.

MEDLINE:93241941

**Cell-specific expression of helix-loop-helix transcription factors encoded by the E2A gene.**

The E2A gene encodes transcription factors of the helix-loop-helix family that are implicated in cell-specific gene expression as part of dimeric complexes that interact with E box enhancer elements. It has previously been shown that transcripts of the E2A gene can be detected in a wide range of cell types. We have now examined expression of the mouse E2A gene at the protein level using polyclonal antisera directed against distinct portions of the E2A protein to probe blots of cellular extracts. A 73 kDa protein was identified by this analysis: this protein is highly enriched in cell lines of B lymphoid origin as compared to pancreatic beta-cells and fibroblast cells. The detection of this protein selectively in extracts of lymphoid cells correlates with the presence of the E box-binding activity LEF1/BCF1 in these cells; this binding activity was previously shown to be efficiently recognized by antiserum directed against E2A gene products. Transfection of cells with full length E2A cDNA leads to appearance of protein co-migrating with the 73 kDa protein on SDS gel electrophoresis and co-migrating with LEF1/BCF1 on mobility shift analysis. Our results are consistent with the view that the DNA-binding activity LEF1/BCF1 is a homodimer of E2A proteins; the selective appearance of this putative cell-specific transcription factor in B lymphoid cells seems to be attributable, at least in part, to the elevated E2A protein concentrations in these cells.

MEDLINE:93136418

**Interleukin-4 inhibits the lipopolysaccharide-induced expression of c-jun and c-fos messenger RNA and activator protein-1 binding activity in human monocytes.**

We studied the effect of interleukin-4 (IL-4) on the lipopolysaccharide (LPS) induction of two immediate early genes c-fos and c-jun. These genes encode proteins that form the dimeric complex activator protein-1 (AP-1), which is active as a transcriptional factor. Maximal accumulation of either c-fos and c-jun messenger RNA (mRNA) occurred 30 minutes after LPS addition. When cells were treated with IL-4 for 5 hours before LPS activation, both the c-fos and the c-jun mRNA expression was decreased. The inhibition of c-fos and c-jun expression by IL-4 in LPS-treated cells was shown to be due to a lower transcription rate of the c-fos and c-jun genes. IL-4 did not affect the stability of the c-fos and c-jun transcripts. Finally, using electrophoretic mobility shift assays, evidence was obtained that IL-4 inhibits LPS-induced expression of AP-1 protein. These data indicate that IL-4 suppresses the induction of transcription factors in human activated monocytes.

MEDLINE:92408021

**Human immunodeficiency virus type 1 Nef protein inhibits NF-kappa B induction in human T cells.**

Human immunodeficiency virus type 1 (HIV-1) can establish a persistent and latent infection in CD4+ T lymphocytes (W.C.Greene, N.Engl.J. Med.324:308-317, 1991; S.M.Schnittman, M.C.Psallidopoulos, H.C. Lane, L.Thompson, M.Baseler, F.Massari, C.H.Fox, N.P.Salzman, and A.S.Fauci, Science 245:305-308, 1989). Production of HIV-1 from latently infected cells requires host cell activation by T-cell mitogens (T.Folks, D.M.Powell, M.M.Lightfoote, S.Benn, M.A. Martin, and A.S.Fauci, Science 231:600-602, 1986; D.Zagury, J. Bernard, R.Leonard, R.Cheynier, M.Feldman, P.S.Sarin, and R.C. Gallo, Science 231:850-853, 1986). This activation is mediated by the host transcription factor NF-kappa B [G.Nabel and D.Baltimore, Nature (London) 326:711-717, 1987]. We report here that the HIV-1-encoded Nef protein inhibits the induction of NF-kappa B DNA-binding activity by T- cell mitogens. However, Nef does not affect the DNA-binding activity of other transcription factors implicated in HIV-1 regulation, including SP-1, USF, URS, and NF-AT. Additionally, Nef inhibits the induction of HIV-1- and interleukin 2-directed gene expression, and the effect on HIV-1 transcription depends on an intact NF-kappa B-binding site. These results indicate that defective recruitment of NF-kappa B may underlie Nef's negative transcriptional effects on the HIV-1 and interleukin 2 promoters. Further evidence suggests that Nef inhibits NF-kappa B induction by interfering with a signal derived from the T-cell receptor complex.

MEDLINE:93046624

**A novel B cell-derived coactivator potentiates the activation of immunoglobulin promoters by octamer-binding transcription factors.**

A novel B cell-restricted activity, required for high levels of octamer/Oct-dependent transcription from an immunoglobulin heavy chain (IgH) promoter, was detected in an in vitro system consisting of HeLa cell-derived extracts complemented with fractionated B cell nuclear proteins. The factor responsible for this activity was designated Oct coactivator from B cells (OCA-B). OCA-B stimulates the transcription from an IgH promoter in conjunction with either Oct-1 or Oct-2 but shows no significant effect on the octamer/Oct-dependent transcription of the ubiquitously expressed histone H2B promoter and the transcription of USF- and Sp1-regulated promoters. Taken together, our results suggest that OCA-B is a tissue-, promoter-, and factor-specific coactivator and that OCA-B may be a major determinant for B cell-specific activation of immunoglobulin promoters. In light of the evidence showing physical and functional interactions between Oct factors and OCA-B, we propose a mechanism of action for OCA-B and discuss the implications of OCA-B for the transcriptional regulation of other tissue-specific promoters.

MEDLINE:92107162

**The cellular oncogene c-myb can interact synergistically with the Epstein-Barr virus BZLF1 transactivator in lymphoid cells.**

Regulation of replicative functions in the Epstein-Barr virus (EBV) genome is mediated through activation of a virally encoded transcription factor, Z (BZLF1). We have shown that the Z gene product, which binds to AP-1 sites as a homodimer and has sequence similarity to c-Fos, can efficiently activate the EBV early promoter, BMRF1, in certain cell types (i.e., HeLa cells) but not others (i.e., Jurkat cells). Here we demonstrate that the c-myb proto-oncogene product, which is itself a DNA-binding protein and transcriptional transactivator, can interact synergistically with Z in activating the BMRF1 promoter in Jurkat cells (a T-cell line) or Raji cells (an EBV-positive B-cell), whereas the c-myb gene product by itself has little effect. The simian virus 40 early promoter is also synergistically activated by the Z/c-myb combination. Synergistic transactivation of the BMRF1 promoter by the Z/c-myb combination appears to involve direct binding by the Z protein but not the c-myb protein. A 30-bp sequence in the BMRF1 promoter which contains a Z binding site (a consensus AP-1 site) is sufficient to transfer high-level lymphoid-specific responsiveness to the Z/c-myb combination to a heterologous promoter. That the c-myb oncogene product can interact synergistically with an EBV-encoded member of the leucine zipper protein family suggests c-myb is likely to engage in similar interactions with cellularly encoded transcription factors.

MEDLINE:93129248

**Alpha-lipoic acid is a potent inhibitor of NF-kappa B activation in human T cells.**

Acquired immunodeficiency syndrome (AIDS) results from infection with a human immunodeficiency virus (HIV). The long terminal repeat (LTR) region of HIV proviral DNA contains binding sites for nuclear factor kappa B (NF-kappa B), and this transcriptional activator appears to regulate HIV activation. Recent findings suggest an involvement of reactive oxygen species (ROS) in signal transduction pathways leading to NF-kappa B activation. The present study was based on reports that antioxidants which eliminate ROS should block the activation of NF-kappa B and subsequently HIV transcription, and thus antioxidants can be used as therapeutic agents for AIDS. Incubation of Jurkat T cells (1 x 10(6) cells/ml) with a natural thiol antioxidant, alpha-lipoic acid, prior to the stimulation of cells was found to inhibit NF-kappa B activation induced by tumor necrosis factor-alpha (25 ng/ml) or by phorbol 12-myristate 13-acetate (50 ng/ml). The inhibitory action of alpha-lipoic acid was found to be very potent as only 4 mM was needed for a complete inhibition, whereas 20 mM was required for N-acetylcysteine. These results indicate that alpha-lipoic acid may be effective in AIDS therapeutics.

MEDLINE:92407982

**A novel Ets-related transcription factor, Elf-1, binds to human immunodeficiency virus type 2 regulatory elements that are required for inducible trans activation in T cells.**

Human immunodeficiency virus type 1 (HIV-1) and HIV-2 are structurally related retroviruses which both cause AIDS in humans. Although both viruses establish latency in quiescent human-peripheral-blood T cells, the asymptomatic phase of HIV-2 infection may be more prolonged than that of HIV-1. The latent phases of both HIV-1 and HIV-2 infection have been shown to be disrupted by T-cell activation, a process that requires host cell transcription factors. In the case of HIV-1, the transcription factor NF-kappa B is sufficient for inducible transcriptional activation. In contrast, factors in addition to NF-kappa B are required to activate HIV-2 transcription in infected T cells. In this report, we demonstrate that a novel Ets-related transcription factor, Elf-1, binds specifically to two purine-rich motifs in the HIV-2 enhancer. Mutagenesis experiments demonstrated that these Elf-1 binding sites are required for induction of HIV-2 transcription following T-cell-receptor-mediated T-cell activation. Moreover, Elf-1 is the only factor present in activated T-cell nuclear extracts that binds to these sites in electrophoretic mobility shift assays. Thus, Elf-1 is a novel transcription factor that appears to be required for the T-cell-receptor-mediated trans activation of HIV-2 gene expression. These results may explain differences in the clinical spectra of diseases caused by HIV-1 and HIV-2 and may also have implications for the design of therapeutic approaches to HIV-2 infection.

MEDLINE:92392375

**Okadaic acid is a potent inducer of AP-1, NF-kappa B, and tumor necrosis factor-alpha in human B lymphocytes.**

Treatment of human B lymphocytes with an optimal concentration of okadaic acid, an inhibitor of phosphatases 1 and 2A, resulted in the induction of the transcription factor, AP-1 and a marked increase in NF-kappa B levels. In contrast, no effect on the levels of the octamer binding proteins, Oct-1 or Oct-2, were found. Since both AP-1 and NF-kappa B have been reported to be important in the induction of the tumor necrosis factor-alpha (TNF-alpha) gene we examined the effects of okadaic acid on TNF-alpha mRNA levels. Treatment with okadaic acid resulted in a striking increase in TNF-alpha mRNA transcripts within 1 h of stimulation and large amounts of TNF-alpha were released into the culture media. Although okadaic acid provides a potent inductive signal for AP-1 and NF-kappa B it did not induce either B cell proliferation or immunoglobulin secretion.

MEDLINE:92317168

**Heterodimerization and transcriptional activation in vitro by NF-kappa B proteins.**

The NF-kappa B family of transcription proteins represents multiple DNA binding, rel related polypeptides that contribute to regulation of genes involved in immune responsiveness and inflammation, as well as activation of the HIV long terminal repeat. In this study multiple NF-kappa B related polypeptides ranging from 85 to 45 kDa were examined for their capacity to interact with the PRDII regulatory element of interferon beta and were shown to possess distinct intrinsic DNA binding affinities for this NF-kappa B site and form multiple DNA binding homo- and heterodimer complexes in co-renaturation experiments. Furthermore, using DNA templates containing two copies of the PRDII domain linked to the rabbit beta globin gene, the purified polypeptides specifically stimulated NF-kappa B dependent transcription in an in vitro reconstitution assay as heterodimers but not as p50 homodimers. These experiments emphasize the role of NF-kappa B dimerization as a distinct level of transcriptional control that may permit functional diversification of a limited number of regulatory proteins.

MEDLINE:92176657

**T cell-specific negative regulation of transcription of the human cytokine IL-4.**

IL-4 secreted by activated T cells is a pleiotropic cytokine affecting growth and differentiation of diverse cell types such as T cells, B cells, and mast cells. We investigated the upstream regulatory elements of the human IL-4 promoter. A novel T cell-specific negative regulatory element (NRE) composed of two protein-binding sites were mapped in the 5' flanking region of the IL-4 gene: -311CTCCCTTCT-303 (NRE-I) and -288CTTTTTGCTT-TGC-300 (NRE-II). A T cell-specific protein Neg-1 and a ubiquitous protein Neg-2 binding to NRE-I and NRE-II, respectively, were identified. Furthermore, a positive regulatory element was found 45 bp downstream of the NRE. The enhancer activity of the PRE was completely suppressed when the NRE was present. These data suggest that IL-4 promoter activity is normally down-regulated by an NRE via repression of the enhancer positive regulatory element. These data may have implications for the stringent control of IL-4 expression in T cells.

MEDLINE:92164748

**Interleukin 6-induced differentiation of a human B cell line into IgM-secreting plasma cells is mediated by c-fos.**

The role of the protooncogene c-fos in interleukin (IL) 6-induced B cell differentiation was assessed. Treatment of SKW 6.4 cells with IL 6 induced a transient and early stimulation of c-fos sense mRNA expression. The effect appeared within 30 min and returned to basal levels after 2 h. The addition of antisense oligonucleotides to c-fos significantly inhibited IL 6-induced IgM production by SKW 6.4 cells (p less than 0.001), whereas control oligonucleotides had no inhibitory effect. These results indicate that activation of c-fos is involved in IL 6-induced differentiation of SKW 6.4 cells into IgM-secreting cells.

MEDLINE:93041375

**Activation of NF-kappa B by interleukin 2 in human blood monocytes.**

We report here that interleukin 2 (IL-2) acts on human blood monocytes by enhancing binding activity of the transcription factor NF-kappa B to its consensus sequence in the 5' regulatory enhancer region of the IL-2 receptor alpha chain (p55). Similarly, IL-2 activates NF-kappa B in the human monocytic cell line U 937, but not in resting human T-cells. This effect is detectable within 15 min and peaks 1 h after exposure to IL-2. Enhanced NF-kappa B binding activity is followed by functional activation in that inducibility of the IL-2 receptor alpha chain is mediated by enhanced NF-kappa B binding and that a heterologous promoter containing the NF-kappa B consensus sequence (-291 to -245) of the IL-2 receptor alpha chain gene is activated. In addition, IL-2 is capable of increasing transcript levels of the p50 gene coding for the p50 subunit of the NF-kappa B transcription factor, whereas mRNA levels of the p65 NF-kappa B gene remained unchanged.

MEDLINE:92185243

**Human T cell activation through the activation-inducer molecule/CD69 enhances the activity of transcription factor AP-1.**

The induction of the AP-1 transcription factor has been ascribed to the early events leading to T cell differentiation and activation. We have studied the regulation of AP-1 activity in human peripheral blood T lymphocytes stimulated through the activation inducer molecule (AIM)/CD69 activation pathway. Phorbol esters are required to induce AIM/CD69 cell-surface expression as well as for triggering the proliferation of T cells in conjunction with anti-AIM mAb. Mobility shift assays showed that addition of anti-AIM mAb to PMA-treated T lymphocytes markedly enhanced the binding activity of AP-1 to its cognate sequence, the phorbol ester response element. In contrast, anti-AIM mAb did not induce any change in the binding activity of NF-kappa B, a transcription factor whose activity is also regulated by protein kinase C. The increase in AP-1-binding activity was accompanied by the marked stimulation of the transcription of c-fos but not that of c-jun. Blockade of the DNA-binding complexes with an anti-Fos mAb demonstrated a direct participation of c-Fos in the AP-1 complexes induced by anti-AIM mAb. Most of the AP-1 activity could be eliminated when the anti-AIM mAb was added to the culture medium in the presence of cycloheximide, suggesting that de novo protein synthesis is crucial for the induction of AP-1-binding activity. These data provide the evidence that activation of human peripheral blood T cells through the AIM activation pathway regulate the activity of AP-1. Therefore, this pathway appears as a crucial step in the initiation of early T cell activation events.

MEDLINE:92123193

**A novel mitogen-inducible gene product related to p50/p105-NF-kappa B participates in transactivation through a kappa B site.**

A Rel-related, mitogen-inducible, kappa B-binding protein has been cloned as an immediate-early activation gene of human peripheral blood T cells. The cDNA has an open reading frame of 900 amino acids capable of encoding a 97-kDa protein. This protein is most similar to the 105-kDa precursor polypeptide of p50-NF-kappa B. Like the 105-kDa precursor, it contains an amino-terminal Rel-related domain of about 300 amino acids and a carboxy-terminal domain containing six full cell cycle or ankyrin repeats. In vitro-translated proteins, truncated downstream of the Rel domain and excluding the repeats, bind kappa B sites. We refer to the kappa B-binding, truncated protein as p50B by analogy with p50-NF-kappa B and to the full-length protein as p97. p50B is able to form heteromeric kappa B-binding complexes with RelB, as well as with p65 and p50, the two subunits of NF-kappa B. Transient-transfection experiments in embryonal carcinoma cells demonstrate a functional cooperation between p50B and RelB or p65 in transactivation of a reporter plasmid dependent on a kappa B site. The data imply the existence of a complex family of NF-kappa B-like transcription factors.

MEDLINE:93110357

**Targeted degradation of c-Fos, but not v-Fos, by a phosphorylation-dependent signal on c-Jun.**

The proto-oncogene products c-Fos and c-Jun heterodimerize through their leucine zippers to form the AP-1 transcription factor. The transcriptional activity of the heterodimer is regulated by signal-dependent phosphorylation and dephosphorylation events. The stability of c-Fos was found to also be controlled by intracellular signal transduction. In transient expression and in vitro degradation experiments, the stability of c-Fos was decreased when the protein was dimerized with phosphorylated c-Jun. c-Jun protein isolated from phorbol ester-induced cells did not target c-Fos for degradation, which suggests that c-Fos is transiently stabilized after stimulation of cell growth. v-Fos protein, the retroviral counterpart of c-Fos, was not susceptible to degradation targeted by c-Jun.

MEDLINE:92378895

**Mitogen stimulation of T-cells increases c-Fos and c-Jun protein levels, AP-1 binding and AP-1 transcriptional activity.**

We have analysed the effect of mitogenic lectins on c-Fos and c-Jun protein levels as well as on activator protein-1 (AP-1) binding and enhancer activity in Jurkat T-cells. Both c-Fos and c-Jun protein levels were increased after Con A and PHA stimulation. Since T-cell stimulation increases both intracellular Ca2+ and cAMP levels and activates protein kinase C (PKC), the possible involvement of these intracellular messengers in c-Fos and c-Jun induction was tested. PMA, which directly activates PKC, mimicked the effect of the lectins on c-Fos and c-Jun, but elevation of either intracellular Ca2+ or cAMP levels had little or no effect. The mitogen-induced increase of c-Fos and c-Jun immunoreactivity was inhibited by H-7, a kinase inhibitor with relatively high specificity for PKC, and less efficiently by H-8, a structurally related kinase inhibitor less active on PKC, but more active on cyclic nucleotide-dependent kinases. Con A stimulation was found to increase both binding of AP-1 to the AP-1 consensus sequence, TRE, and AP-1 enhancer activity, in Jurkat cells. PMA was also found to increase the AP-1 enhancer activity, whereas elevation of Ca2+ or cAMP had only minor effects. We conclude that stimulation with mitogenic lectins is sufficient to increase both c-Fos and c-Jun protein levels, AP-1 binding and AP-1 enhancer activity in Jurkat cells and that they act via mechanisms that could involve the activation of PKC.

MEDLINE:92368408

**SRC-related proto-oncogenes and transcription factors in primary human T cells: modulation by cyclosporin A and FK506.**

Activation of T lymphocytes induces transcription of genes encoding for lymphokines. Interleukin-2 (IL-2) gene expression is controlled transcriptionally by the cooperative activity of specific trans-activating factors that bind to the IL-2 enhancer. Cyclosporin A (CsA) and FK506 inhibit the production of IL-2 in T lymphocytes at the level of gene transcription. A member of the src gene family, the lymphocyte-specific protein tyrosine kinase, p56lck, has been implicated in IL-2 production. CsA was found not to inhibit lck gene expression, nor the activity of the lck gene product. However, CsA and FK506 inhibit the appearance of DNA binding activity of factors that bind to the NF-AT and AP-1 sites in the IL-2 enhancer. Since the induction of NF-AT and AP-1 is induced by the same stimuli that stimulate IL-2 production, these results indicate that the immunosuppressant action of CsA and FK506 is exerted at the level of these trans-activating factors.

MEDLINE:92156807

**The AP-1 site at -150 bp, but not the NF-kappa B site, is likely to represent the major target of protein kinase C in the interleukin 2 promoter.**

Stimulation of T cells with antigen results in activation of several kinases, including protein kinase C (PKC), that may mediate the later induction of activation-related genes. We have examined the potential role of PKC in induction of the interleukin 2 (IL-2) gene in T cells stimulated through the T cell receptor/CD3 complex. We have previously shown that prolonged treatment of the untransformed T cell clone Ar-5 with phorbol esters results in downmodulation of the alpha and beta isozymes of PKC, and abrogates induction of IL-2 mRNA and protein. Here we show that phorbol ester treatment also abolishes induction of chloramphenicol acetyltransferase activity in Ar-5 cells transfected with a plasmid containing the IL-2 promoter linked to this reporter gene. The IL-2 promoter contains binding sites for nuclear factors including NFAT-1, Oct, NF-kappa B, and AP-1, which are all potentially sensitive to activation of PKC. We show that induction of a trimer of the NFAT and Oct sites is not sensitive to phorbol ester treatment, and that mutations in the NF-kappa B site have no effect on inducibility of the IL-2 promoter. In contrast, mutations in the AP-1 site located at -150 bp almost completely abrogate induction of the IL-2 promoter, and appearance of an inducible nuclear factor binding to this site is sensitive to PKC depletion. Moreover, cotransfections with c-fos and c-jun expression plasmids markedly enhance induction of the IL-2 promoter in minimally stimulated T cells. Our results indicate that the AP-1 site at -150 bp represents a major, if not the only, site of PKC responsiveness in the IL-2 promoter.

MEDLINE:93153017

**I kappa B/MAD-3 masks the nuclear localization signal of NF-kappa B p65 and requires the transactivation domain to inhibit NF-kappa B p65 DNA binding.**

The active nuclear form of the NF-kappa B transcription factor complex is composed of two DNA binding subunits, NF-kappa B p65 and NF-kappa B p50, both of which share extensive N-terminal sequence homology with the v-rel oncogene product. The NF-kappa B p65 subunit provides the transactivation activity in this complex and serves as an intracellular receptor for a cytoplasmic inhibitor of NF-kappa B, termed I kappa B. In contrast, NF-kappa B p50 alone fails to stimulate kappa B-directed transcription, and based on prior in vitro studies, is not directly regulated by I kappa B. To investigate the molecular basis for the critical regulatory interaction between NF-kappa B and I kappa B/MAD-3, a series of human NF-kappa B p65 mutants was identified that functionally segregated DNA binding, I kappa B-mediated inhibition, and I kappa B-induced nuclear exclusion of this transcription factor. Results from in vivo expression studies performed with these NF-kappa B p65 mutants revealed the following: 1) I kappa B/MAD-3 completely inhibits NF-kappa B p65-dependent transcriptional activation mediated through the human immunodeficiency virus type 1 kappa B enhancer in human T lymphocytes, 2) the binding of I kappa B/MAD-3 to NF-kappa B p65 is sufficient to retarget NF-kappa B p65 from the nucleus to the cytoplasm, 3) selective deletion of the functional nuclear localization signal present in the Rel homology domain of NF-kappa B p65 disrupts its ability to engage I kappa B/MAD-3, and 4) the unique C-terminus of NF-kappa B p65 attenuates its own nuclear localization and contains sequences that are required for I kappa B-mediated inhibition of NF-kappa B p65 DNA binding activity. Together, these findings suggest that the nuclear localization signal and transactivation domain of NF-kappa B p65 constitute a bipartite system that is critically involved in the inhibitory function of I kappa B/MAD-3. Unexpectedly, our in vivo studies also demonstrate that I kappa B/MAD-3 binds directly to NF-kappa B p50. This interaction is functional as it leads to retargeting of NF-kappa B p50 from the nucleus to the cytoplasm. However, no loss of DNA binding activity is observed, presumably reflecting the unique C-terminal domain that is distinct from that present in NF-kappa B p65.

MEDLINE:93024882

**The candidate oncoprotein Bcl-3 is an antagonist of p50/NF-kappa B-mediated inhibition.**

The candidate oncogene bcl-3 was discovered as a translocation into the immunoglobulin alpha-locus in some cases of B-cell chronic lymphocytic leukaemias. The protein Bcl-3 contains seven so-called ankyrin repeats. Similar repeat motifs are found in a number of diverse regulatory proteins but the motifs of Bcl-3 are most closely related to those found in I kappa B proteins in which the ankyrin repeat domain is thought to be directly involved in inhibition of NF-kappa B activity. No biological function has yet been described for Bcl-3, but it was noted recently that Bcl-3 interferes with DNA-binding of the p50 subunit of NF-kappa B in vitro. Here we demonstrate that Bcl-3 can aid kappa B site-dependent transcription in vivo by counteracting the inhibitory effects of p50/NF-kappa B homodimers. Bcl-3 may therefore aid activation of select NF-kappa B-regulated genes, including those of the human immunodeficiency virus.

MEDLINE:92244343

**Nuclear factor of activated T cells contains Fos and Jun.**

The nuclear factor NF-AT (ref. 1) is induced in T cells stimulated through the T-cell receptor/CD3 complex, and is required for interleukin-2 (IL-2) gene induction. Although NF-AT has not been cloned or purified, there is evidence that it is a major target for immunosuppression by cyclosporin A (CsA) and FK506 (refs 2-7). NF-AT induction may require two activation-dependent events: the CsA-sensitive translocation of a pre-existing component and the CsA-resistant synthesis of a nuclear component. Here we report that the newly synthesized nuclear component of NF-AT is the transcription factor AP-1. We show that the inducible nuclear form of NF-AT contains Fos and Jun proteins. Furthermore, we identify a pre-existing NF-AT-binding factor that is present in hypotonic extracts of unstimulated T cells. On the basis of binding, reconstitution and cotransfection experiments, we propose that activation of NF-AT occurs in at least two stages: a CsA-sensitive stage involving modification and/or translocation of the pre-existing NF-AT complex, and a CsA-insensitive stage involving the addition of newly synthesized Fos or Fos/Jun proteins to the pre-existing complex.

MEDLINE:92195320

**A lymphoid cell-specific nuclear factor containing c-Rel-like proteins preferentially interacts with interleukin-6 kappa B-related motifs whose activities are repressed in lymphoid cells.**

The proto-oncoprotein c-Rel is a member of the nuclear factor kappa B transcription factor family, which includes the p50 and p65 subunits of nuclear factor kappa B. We show here that c-Rel binds to kappa B sites as homodimers as well as heterodimers with p50. These homodimers and heterodimers show distinct DNA-binding specificities and affinities for various kappa B motifs. In particular, the c-Rel homodimer has a high affinity for interleukin-6 (IL-6) and beta interferon kappa B sites. In spite of its association with p50 in vitro, however, we found a lymphoid cell-specific nuclear factor in vivo that contains c-Rel but not p50 epitopes; this factor, termed IL-6 kappa B binding factor II, appears to contain the c-Rel homodimer and preferentially recognizes several IL-6 kappa B-related kappa B motifs. Although it has been previously shown that the IL-6 kappa B motif functions as a potent IL-1/tumor necrosis factor-responsive element in nonlymphoid cells, its activity was found to be repressed in lymphoid cells such as a Jurkat T-cell line. We also present evidence that IL-6 kappa B binding factor II functions as a repressor specific for IL-6 kappa B-related kappa B motifs in lymphoid cells.

MEDLINE:94051535

**Cellular immune and cytokine pathways resulting in tissue factor expression and relevance to septic shock.**

Cells of monocyte lineage serve as effector cells in the cellular immune response. In addition, they respond to LPS and cytokines with activation and expression of inflammatory effector gene products similar to those elicited by the antigen driven response. The response to antigen proceeds at the T helper cell level through two independent forms of cellular collaboration, contact and lymphokine. We review the control of expression of the Tissue Factor (TF) gene and the function of the TF protein. The enhanced initiation of transcription of the TF gene appears to require engagement of a 56 bp LPS Response Element, an enhancer that is engaged by both AP-1 type heterodimeric complexes as well as NF kappa B like heterodimeric complexes. Dissociation of NF kappa B from Ig kappa B by cytokine and LPS stimulation, and possibly activated T cells, may represent a common pathway to induction of the TF and other inflammatory genes. Enhancement of expression of TF is observed upon adhesion of Mo to endothelial cells and extracellular matrix proteins, as well as upon engagement of leukocyte integrins. The biological effects that follow from expression of TF by vascular cells have been resolved by analysis of function aided by the use of recombinant full length TF and truncated surface domain of TF. The rules of assembly of the cognate ligands of TF, namely the zymogen plasma factors VII and the serine protease factor VIIa, with the soluble surface domain of TF in free solution, in the presence of phospholipid surfaces and cell surface and of the anchored TF molecule have been described. It is evident that assembly of the surface domain of TF with VIIa to form the binary TF.VIIa complex induces a significant increase in the Kcat of the catalytic domain of VIIa for small peptidyl substrates and more profoundly for protein substrate. This provides substantial evidence for an allosteric effect on the catalytic cleft of VIIa that is imparted by binding to TF, its cognate catalytic cofactor. It is also evident that the TF.VIIa complex is proteolytically active and can activate the zymogen plasma factor X to the serine protease Xa in free solution, inferring that extended substrate recognition by induced structural loci of the TF.VIIa complex are created from either or both proteins to constitute a new recognition structure. It is also evident that association of X with charged phospholipid surfaces enhances the proteolytic activation of this zymogen by increasing recognition and susceptibility of the sessile peptide bond deduced from the markedly decreased Km and increased Kcat.

MEDLINE:92260664

NF-kappa B is a protein complex which functions in concert with the tat-I gene product to stimulate human immunodeficiency virus (HIV) transcription. To determine whether specific members of the NF-kappa B family contribute to this effect, we have examined the abilities of different NF-kappa B subunits to act with Tat-I to stimulate transcription of HIV in Jurkat T-leukemia cells. We have found that the p49(100) DNA binding subunit, together with p65, can act in concert with Tat-I to stimulate the expression of HIV-CAT plasmid. Little effect was observed with 50-kDa forms of p105 NF-kappa B or rel, in combination with p65 or full-length c-rel, which do not stimulate the HIV enhancer in these cells. These findings suggest that the combination of p49(100) and p65 NF-kappa B can act in concert with the tat-I gene product to stimulate the synthesis of HIV RNA.

MEDLINE:93065212

**Characterization of a novel T lymphocyte protein which binds to a site related to steroid/thyroid hormone receptor response elements in the negative regulatory sequence of the human immunodeficiency virus long terminal repeat.**

We have previously identified a T lymphocyte protein which binds to a site within the LTR of the human immunodeficiency virus type 1 (HIV-1) and exerts an inhibitory effect on virus gene expression. The palindromic site (site B) recognized by this protein is related to the palindromic binding sites of members of the steroid/thyroid hormone receptor family. Here we characterize the T cell protein binding to this site as a 100 kD protein which is most abundant in T cells and which binds to site B as a 200 kD complex. This protein is distinct from other members of the steroid/thyroid hormone receptor family including the COUP protein which has a closely related DNA binding specificity.

MEDLINE:92260679

**Activation of the human immunodeficiency virus type 1 enhancer is not dependent on NFAT-1.**

The function of a putative NFAT-1 site in the human immunodeficiency virus type 1 enhancer has been analyzed. Activation by the T-cell antigen receptor is minimal in Jurkat cells and is mediated by the kappa B sites. The putative NFAT-1 region is not required for the response to anti-CD3 or to mitogens in T-cell, B-cell, or monocyte/macrophage leukemia lines, nor is it a cis-acting negative regulatory element.

MEDLINE:92218428

**The B cell-specific nuclear factor OTF-2 positively regulates transcription of the human class II transplantation gene, DRA.**

The promoter of the major histocompatibility class II gene DRA contains an octamer element (ATTTGCAT) that is required for efficient DRA expression in B cells. Several DNA-binding proteins are known to bind this sequence. The best characterized are the B cell-specific OTF-2 and the ubiquitous OTF-1. This report directly demonstrates that OTF-2 but not OTF-1 regulates the DRA gene. In vitro transcription analysis using protein fractions enriched for the octamer-binding protein OTF-2 demonstrate a positive functional role for OTF-2 in DRA gene transcription. In contrast, OTF-1-enriched protein fractions did not affect DRA gene transcription although it functionally enhanced the transcription of another gene. Recombinant OTF-2 protein produced by in vitro transcription/translation could also enhance DRA gene transcription in vitro. In vivo transient transfection studies utilizing an OTF-2 expression vector resulted in similar findings: that OTF-2 protein enhanced DRA gene transcription, and that this effect requires an intact octamer element. Together these results constitute the first direct evidence of a positive role for the lymphoid-specific octamer-binding factor in DRA gene transcription.

MEDLINE:92251244

**Interferon-gamma potentiates the antiviral activity and the expression of interferon-stimulated genes induced by interferon-alpha in U937 cells.**

Binding of type I interferon (IFN-alpha/beta) to specific receptors results in the rapid transcriptional activation, independent of protein synthesis, of IFN-alpha-stimulated genes (ISGs) in human fibroblasts and HeLa and Daudi cell lines. The binding of ISGF3 (IFN-stimulated gene factor 3) to the conserved IFN-stimulated response element (ISRE) results in transcriptional activation. This factor is composed of a DNA-binding protein (ISGF3 gamma), which normally is present in the cytoplasm, and other IFN-alpha-activated proteins which preexist as latent cytoplasmic precursors (ISGF3 alpha). We have found that ISG expression in the monocytic U937 cell line differs from most cell lines previously examined. U937 cells express both type I and type II IFN receptors, but only IFN-alpha is capable of inducing antiviral protection in these cells. Pretreatment with IFN-gamma potentiates the IFN-alpha-induced protection, but IFN-gamma alone does not have any antiviral activity. ISG15 mRNA accumulation in U937 cells is not detectable before 6 h of IFN-alpha treatment, peaks at 24 h, and requires protein synthesis. Although IFN-gamma alone does not induce ISG expression, IFN-gamma pretreatment markedly increases and hastens ISG expression and transcriptional induction. Nuclear extracts assayed for the presence of ISRE binding factors by electrophoretic mobility shift assays show that ISGF3 is induced by IFN-alpha within 6 h from undetectable basal levels in untreated U937 cells. Activation of ISGF3 alpha, the latent component of ISGF3, occurs rapidly. However, the increase in ISGF3 activity ultimately correlates with the accumulation of ISGF3 gamma induced by IFN-alpha or IFN-gamma. (ABSTRACT TRUNCATED AT 250 WORDS)

MEDLINE:92224250

**Transcription factor activation and functional stimulation of human monocytes.**

Activation of expression of genes encoding transcription factors: c-fos and c-jun and formation of AP1 transcriptional complex in human monocytes was investigated. It was found that lipopolysaccharide induced strongly both c-fos and c-jun expression as well as AP1 formation. Interferon gamma activated strongly c-fos and weakly c-jun and AP1. Tumor necrosis factor induced slightly c-fos and had almost no effect on c-jun and AP1. The data suggest that differences in functional responses elicited in monocytes by all three factors may be dependent on different routes on nuclear signalling employed by the factors.

MEDLINE:92062170

**Regulation of interleukin-1 beta production by glucocorticoids in human monocytes: the mechanism of action depends on the activation signal.**

Glucocorticoids are known to downregulate interleukin-1 beta production in monocytic cells by two different mechanims: direct inhibition of the gene transcription and destabilization of the preformed interleukin-1 beta mRNA. Now we have examined the effect of the nature of the monocyte activating signal on these two inhibitory mechanims. When human monocytes were preincubated with dexamethasone for 1 hour and then stimulated either with bacterial lipopolysaccharide or phorbol myristate, it was found that dexamethasone inhibited the lipopolysaccharide-induced interleukin-1 beta protein production, but the phorbol myristate-induced production was increased 3-10 fold. This difference was also seen at the mRNA level. When dexamethasone was added to the cultures 3 hours after the stimulators, it clearly decreased the interleukin-1 beta mRNA levels regardless of the stimulator used (although the effect was clearly weaker on the PMA-induced mRNA). Thus these data suggest that the phorbol myristate-induced signal (prolonged protein kinase C activation?) cannot be inhibited by prior incubation with dexamethasone and it also protects the induced mRNA for the degradative action of dexamethasone.

MEDLINE:92001749

**A nuclear factor NF-GM2 that interacts with a regulatory region of the GM-CSF gene essential for its induction in responses to T-cell activation: purification from human T-cell leukemia line Jurkat cells and similarity to NF-kappa B.**

Activation of T cells by antigen, lectin, or a combination of phorbol-12-myristate acetate (PMA) and calcium ionophore (A23187) leads to the induction of genes for a set of lymphokines, including granulocyte-macrophage colony-stimulating factor (GM-CSF). We demonstrated in earlier studies that the upstream region of the mouse GM-CSF promoter at positions between -95 and -73 is essential for transcriptional activation in response to PMA/A23187. This region contains two DNA-binding motifs, GM2 and GC-box. The GM2 sequence (GGTAGTTCCC) is recognized by an inducible factor NF-GM2; the other (CCGCCC) by constitutive factors A1, A2, and B. To elucidate the mechanism of GM-CSF gene activation, we have purified the inducible factor NF-GM2 from the nuclear extract of stimulated Jurkat cells on the basis of specific DNA-binding activity. The purified NF-GM2 consists of 50 (p50) and 65 kDa (p65) polypeptides and has a binding activity specific for both the GM-CSF and immunoglobulin kappa (GGAAAGTCCC) enhancers. Electrophoretically purified p50 alone can form a protein-DNA complex, but in the mixture, p50 associates preferentially with p65 to form the NF-GM2 complex. In addition, p65 gave per se, with low affinity, a protein-DNA complex that migrated more slowly than native NF-GM2 complex. Furthermore, an antiserum against KBF1 (identical to 50 kDa NF-kappa B protein) reacted with the p50 of NF-GM2, indicating that the NF-GM2 polypeptide cannot be immunologically differentiated from the 50 kDa subunit of NF-kappa B. The purified NF-GM2 activated in vitro transcription from the kappa B enhancer, while it failed to stimulate transcription from the GM-CSF promoter harboring the GM2 sequence. This suggests that the activation mechanism of the GM-CSF gene through the GM2/GC-box sequence is different from that of genes carrying the kappa B enhancer alone.

MEDLINE:91292530

**Characterization of an immediate-early gene induced in adherent monocytes that encodes I kappa B-like activity.**

We have cloned a group of cDNAs representing mRNAs that are rapidly induced following adherence of human monocytes. One of the induced transcripts (MAD-3) encodes a protein of 317 amino acids with one domain containing five tandem repeats of the cdc10/ankyrin motif, which is 60% similar (46% identical) to the ankyrin repeat region of the precursor of NF-kappa B/KBF1 p50. The C-terminus has a putative protein kinase C phosphorylation site. In vitro translated MAD-3 protein was found to specifically inhibit the DNA-binding activity of the p50/p65 NF-kappa B complex but not that of the p50/p50 KBF1 factor or of other DNA-binding proteins. The MAD-3 cDNA encodes an I kappa B-like protein that is likely to be involved in regulation of transcriptional responses to NF-kappa B, including adhesion-dependent pathways of monocyte activation.

MEDLINE:91173312

**Isolation of a rel-related human cDNA that potentially encodes the 65-kD subunit of NF-kappa B [published erratum appears in Science 1991 Oct 4;254(5028):11]**

A DNA probe that spanned a domain conserved among the proto-oncogene c-rel, the Drosophila morphogen dorsal, and the p50 DNA binding subunit of NF-kappa B was generated from Jurkat T cell complementary DNA with the polymerase chain reaction (PCR) and degenerate oligonucleotides. This probe was used to identify a rel-related complementary DNA that hybridized to a 2.6-kilobase messenger RNA present in human T and B lymphocytes. In vitro transcription and translation of the complementary DNA resulted in the synthesis of a protein with an apparent molecular size of 65 kilodaltons (kD). The translated protein showed weak DNA binding with a specificity for the kappa B binding motif. This protein-DNA complex comigrated with the complex obtained with the purified human p65 NF-kappa B subunit and binding was inhibited by I kappa B-alpha and -beta proteins. In addition, the 65-kD protein associated with the p50 subunit of NF-kappa B and the kappa B probe to form a complex with the same electrophoretic mobility as the NF-kappa B-DNA complex. Therefore the rel-related 65-kD protein may represent the p65 subunit of the active NF-kappa B transcription factor complex.

MEDLINE:91354520

**Regulation of M-CSF expression by M-CSF: role of protein kinase C and transcription factor NF kappa B.**

Macrophage-colony-stimulating factor (M-CSF), also referred to as CSF-1, regulates the survival, growth, differentiation and functional activity of monocytes by binding to a single class of high-affinity cell surface receptors, known to be the product of the c-fms protooncogene. The detection of both M-CSF and c-fms expression by cells of the monocyte lineage has suggested that M-CSF may act by an autocrine mechanism. Interestingly, it has been shown that M-CSF can induce the expression of its own gene. Although sensitivity to M-CSF can be modulated by regulation of receptor expression and function, M-CSF responsiveness is largely determined at a postreceptor level. To date, little is known about the intracellular pathway of M-CSF signal transduction. We have therefore investigated the changes in protein kinase C (PKC) activity upon exposure of monocytes to M-CSF. We show that M-CSF activates and translocates PKC. Inhibition of PKC by the isoquinoline derivative H7 abolishes induction of M-CSF by M-CSF. Furthermore, activation of PKC was pertussis-toxin-sensitive and was associated with the detection of an NF kappa B protein in nuclear extracts of M-CSF-induced blood monocytes but not in monocytes exposed to medium treatment only. The results suggest that M-CSF induction of M-CSF involves G proteins, PKC and NF kappa B.

MEDLINE:92013023

**Anti-CD2 receptor antibodies activate the HIV long terminal repeat in T lymphocytes.**

The CD2 T lymphocyte glycoprotein surface molecule mediates both cell to cell adhesion and T cell activation, two processes that are involved in the spread of HIV infection. Treatment of chronically HIV-infected PBMC with anti-CD2 mAb has been shown to induce the expression of infectious virus from these cultures. In this study we investigated the mechanisms whereby anti-CD2 antibodies stimulate viral production. We demonstrate that treatment of transiently transfected T lymphocytes with anti-CD2 antibodies results in activation of the HIV long terminal repeat. Furthermore, CAT assays using mutated HIV long terminal repeat-CAT constructs and gel shift assays demonstrate that this activation is dependent on the NF-kappa B enhancer. These studies suggest that interaction of CD2 with its natural ligand, LFA-3, may play a role in regulation of HIV expression.

MEDLINE:91266912

**Inhibition of transcription factors belonging to the rel/NF-kappa B family by a transdominant negative mutant.**

The KBF1 factor, which binds to the enhancer A located in the promoter of the mouse MHC class I gene H-2Kb, is indistinguishable from the p50 DNA binding subunit of the transcription factor NF-kappa B, which regulates a series of genes involved in immune and inflammatory responses. The KBF1/p50 factor binds as a homodimer but can also form heterodimers with the products of other members of the same family, like the c-rel and v-rel (proto)oncogenes. The dimerization domain of KBF1/p50 is contained between amino acids 201 and 367. A mutant of KBF1/p50 (delta SP), unable to bind to DNA but able to form homo- or heterodimers, has been constructed. This protein reduces or abolishes in vitro the DNA binding activity of wild-type proteins of the same family (KBF1/p50, c- and v-rel). This mutant also functions in vivo as a trans-acting dominant negative regulator: the transcriptional inducibility of the HIV long terminal repeat (which contains two potential NF-kappa B binding sites) by phorbol ester (PMA) is inhibited when it is co-transfected into CD4+ T cells with the delta SP mutant. Similarly the basal as well as TNF or IL1-induced activity of the MHC class I H-2Kb promoter can be inhibited by this mutant in two different cell lines. These results constitute the first formal demonstration that these genes are regulated by members of the rel/NF-kappa B family.

MEDLINE:92051383

**One base pair change abolishes the T cell-restricted activity of a kB-like proto-enhancer element from the interleukin 2 promoter.**

The inducible, T cell-specific enhancers of murine and human Interleukin 2 (Il-2) genes contain the kB-like sequence GGGATTTCACC as an essential cis-acting enhancer motif. When cloned in multiple copies this so-called TCEd (distal T cell element) acts as an inducible proto-enhancer element in E14 T lymphoma cells, but not in HeLa cells. In extracts of induced, Il-2 secreting El4 cells three individual protein factors bind to TCEd DNA. The binding of the most prominent factor, named TCF-1 (T cell factor 1), is correlated with the proto-enhancer activity of TCEd. TCF-1 consists of two polypeptides of about 50 kD and 105 kD; the former seems to be related to the 50 kD polypeptide of NF-kB. Purified NF-kB is also able to bind to the TCEd, but TCF-1 binds stronger than NF-kB to TCEd DNA. The conversion of the TCEd to a 'perfect' NF-kB binding site leads to a tighter binding of NF-kB to TCEd DNA and, as a functional consequence, to the activity of the 'converted' TCEd motifs in HeLa cells. Thus, the substitution of the underlined A residue to a C within the GGGATTTCACC motif abolishes its T cell-restricted activity and leads to its functioning in both El4 cells and HeLa cells. These results indicate that lymphocyte-specific factors binding to the TCEd are involved in the control of T cell specific-transcription of the Il-2 gene.

MEDLINE:91296379

**HTLV-1 Tax induces expression of various immediate early serum responsive genes.**

Human T-cell leukemia virus type 1 (HTLV-1) is an etiological agent of adult T-cell leukemia (ATL). We showed here by mobility-shift assay that T-cell lines transformed with the virus contained high levels of AP-1 activities. Consistent with this result, these cell lines expressed increased levels of mRNAs encoding the AP-1 proteins, c-Fos, Fra-1, c-Jun, JunB, and JunD. Previously, transcription of the c-fos gene has been reported to be transactivated by the viral transcription factor, Tax1. By using the human T-cell line (JPX-9), in which expression of the Tax1 is inducible, we showed that expression of mRNAs for Fra-1, c-Jun, and JunD was also transactivated by Tax1. Moreover, Tax1 activated expression of two other transcription factors having zinc finger motifs, Egr-1 and Egr-2, in the same cells. The Tax1-inducible transcription factors identified here are encoded by the members of immediate early genes under the control of growth signals. Thus, Tax1 was suggested to replace growth signals, at least in part, by this mechanism.

MEDLINE:91210272

**Vitamin D receptor expression in human lymphocytes. Signal requirements and characterization by western blots and DNA sequencing.**

The signals controlling the expression of the receptor protein for 1 alpha,25-dihydroxyvitamin D3 in normal human lymphocytes and the relationship of this protein to the classical vitamin D receptor were examined. Lymphocytes activated with the OKT3 antibody to the T-cell antigen receptor expressed fewer binding sites as compared to lymphocytes that were activated by the polyclonal activator phytohemagglutinin (PHA). However, combination of OKT3 and phorbol myristate acetate produced a concentration of binding sites similar to the PHA-activated cells. The receptor from OKT3 and OKT3 + phorbol myristate acetate-activated lymphocytes exhibited decreased binding to DNA-cellulose compared to PHA-activated lymphocytes. In lymphocytes activated either by PHA or OKT3 (but not in resting cells), a 50-kDa species cross-reacting with a monoclonal antibody against the intestinal vitamin D receptor was detected. Finally, RNA from activated lymphocytes was amplified by polymerase chain reaction using oligonucleotide primers flanking the 196 base pair long region encoding the DNA-binding domain of the human intestinal receptor. The amplified product showed an identical nucleotide sequence to the DNA-binding domain of the human intestinal receptor. These findings suggest that expression of the 1,25-(OH)2D3 receptor in lymphocytes is triggered by distinct and contingent signals, and that the protein and the mRNA encoding it are identical to the classical vitamin D receptor.

MEDLINE:91110562

**Specific depletion of the B-cell population induced by aberrant expression of human interferon regulatory factor 1 gene in transgenic mice.**

Interferons (IFNs) are well known both as antiviral proteins and as potent regulators of cell growth and differentiation. In fact, IFNs inhibit growth of various normal and transformed cell types. Previously, a nuclear factor, IRF-1 (interferon regulatory factor 1), which binds to type I IFN and some IFN-inducible gene promoters, was identified and cloned. Since the IRF-1 gene is both virus and IFN inducible, an intriguing issue is raised as to whether the IRF-1 gene is functioning in IFN-mediated regulation of cell growth and differentiation. In this study, we generated transgenic mice carrying the human IRF-1 gene linked to the human immunoglobulin heavy-chain enhancer. In the transgenic mice, all the lymphoid tissues examined showed a dramatic reduction in the number of B lymphocytes (B cells). Preparation and analysis of bone marrow cells from the chimeric mice indicated that the bone marrow is the effective site for specific depletion of the B-cell population. In fact, transgenic bone marrow cells cocultured with a bone marrow-derived stromal cell line revealed an altered B-cell maturation pattern.

MEDLINE:91237114

**Transforming growth factor-beta suppresses human B lymphocyte Ig production by inhibiting synthesis and the switch from the membrane form to the secreted form of Ig mRNA.**

Transforming growth factor-beta (TGF-beta) inhibits B cell Ig secretion and reduces B cell membrane Ig expression. The addition of TGF-beta to human B lymphocyte cultures stimulated with Staphylococcus aureus Cowan strain I and IL-2 completely inhibited B cell Ig secretion (greater than 90%) and decreased B cell surface IgM, IgD, kappa L chain, and lambda L chain expression. In contrast, TGF-beta had only minimal effects on two other B cell membrane proteins, HLA-DR and CD20. Internal labeling with [35S]methionine and immunoprecipitation with anti-IgM, anti-kappa, and anti-lambda antibodies revealed a striking reduction in kappa L chain in the presence of TGF-beta. A less pronounced reduction in lambda L chain and microH chain was also noted. Northern blot analysis of RNA purified from B cells treated with TGF-beta for varying time intervals revealed a significant decrease in steady state kappa and lambda L chain mRNA levels. Furthermore, a significant decrease in the switch from the membrane forms of mu and gamma to their respective secreted forms was noted in the presence of TGF-beta. Nuclear run-on experiments demonstrated decreased transcription of kappa L chain. The effects of TGF-beta on two transcriptional regulatory factors, Oct-2 and nuclear factor (NF) kappa B, known to be important in Ig gene transcription were examined. Oct-2 mRNA levels and both Oct-2 and NF-kappa B proteins in nuclear extracts were not altered by treatment with TGF-beta. In contrast, levels of the transcriptional factor AP-1, which is not known to be important in B cell Ig production, were reduced by TGF-beta. These findings demonstrate that TGF-beta decreases B lymphocyte Ig secretion by inhibiting the synthesis of Ig mRNA and inhibiting the switch from the membrane form to the secreted forms of mu and gamma mRNA. The mechanism by which TGF-beta inhibits Ig chain synthesis is unclear although it does not involve inhibition of the binding of NF-kappa B or Oct-2 to their respective target sequences.

MEDLINE:92052179

**Isolation of a candidate repressor/activator, NF-E1 (YY-1, delta), that binds to the immunoglobulin kappa 3' enhancer and the immunoglobulin heavy-chain mu E1 site.**

We have determined that the developmental control of immunoglobulin kappa 3' enhancer (kappa E3') activity is the result of the combined influence of positive- and negative-acting elements. We show that a central core in the kappa E3' enhancer is active at the pre-B-cell stage but is repressed by flanking negative-acting elements. The negative-acting sequences repress enhancer activity in a position- and orientation-independent manner at the pre-B-cell stage. We have isolated a human cDNA clone encoding a zinc finger protein (NF-E1) that binds to the negative-acting segment of the kappa E3' enhancer. This protein also binds to the immunoglobulin heavy-chain enhancer mu E1 site. NF-E1 is encoded by the same gene as the YY-1 protein, which binds to the adeno-associated virus P5 promoter. NF-E1 is also the human homologue of the mouse delta protein, which binds to ribosomal protein gene promoters. The predicted amino acid sequence of this protein contains features characteristic of transcriptional activators as well as transcriptional repressors. Cotransfection studies with this cDNA indicate that it can repress basal promoter activity. The apparent dual function of this protein is discussed.

MEDLINE:91367666

**USF-related transcription factor, HIV-TF1, stimulates transcription of human immunodeficiency virus-1.**

The transcription factor HIV-TF1, which binds to a region about 60 bp upstream from the enhancer of the human immunodeficiency virus-1 (HIV-1), was purified from human B cells. HIV-TF1 had a molecular weight of 39,000. Binding of HIV-TF1 to the HIV long terminal repeat (LTR) activated transcription from the HIV promoter in vitro. The HIV-TF1-binding site in HIV LTR was similar to the site recognized by upstream stimulatory factor (USF) in the adenovirus major late promoter. DNA-binding properties of HIV-TF1 suggested that HIV-TF1 might be identical or related to USF. Interestingly, treatment of purified HIV-TF1 by phosphatase greatly reduced its DNA-binding activity, suggesting that phosphorylation of HIV-TF1 was essential for DNA binding. The disruption of HIV-TF1-binding site induced a 60% decrease in the level of transcription from the HIV promoter in vivo. These results suggest that HIV-TF1 is involved in transcriptional regulation of HIV-1.

MEDLINE:91237803

**Transactivation of the human immunodeficiency virus promoter by human herpesvirus 6 (HHV-6) strains GS and Z-29 in primary human T lymphocytes and identification of transactivating HHV-6(GS) gene fragments.**

Human herpesvirus 6 (HHV-6) can activate the human immunodeficiency virus (HIV) promoter and accelerate cytopathic effects in HIV-infected human T cells. This study examines the regions of the HIV promoter required for HHV-6 transactivation in a heterogeneous population of primary human T lymphocytes with or without antigenic stimulation. Two different strains of HHV-6, GS and Z29, transactivated the HIV promoter. The GS strain transactivated the promoter in both stimulated and resting T cells, while the Z29 strain increased HIV promoter activity only in stimulated T cells. Three DNA clones containing HHV-6(GS) genomic fragments transactivated the HIV promoter in cotransfected T cells. A 21.4-kb DNA clone, pZVB70, showed the highest transactivating ability, while two other DNA fragments, pZVB10 (6.2 kb) and pZVH14 (8.7 kb), showed lower activity. One of these clones, pZVH14, activated the HIV promoter construct containing a mutation in the NF kappa B site. However, this mutated NF kappa B promoter was not transactivated during HHV-6(GS) infection or after cotransfection with pZVB70 or pZVB10. These data indicate that the NF kappa B sites of the HIV promoter are essential for its transactivation during HHV-6(GS) infection. By increasing HIV promoter activity in primary T lymphocytes, HHV-6 may consequently increase HIV replication, leading to an increase in the cytopathic effect on coinfected human T cells.

MEDLINE:92052106

**Negative regulation of human immunodeficiency virus type 1 expression in monocytes: role of the 65-kDa plus 50-kDa NF-kappa B dimer.**

Although monocytic cells can provide a reservoir for viral production in vivo, their regulation of human immunodeficiency virus type 1 (HIV-1) transcription can be either latent, restricted, or productive. These differences in gene expression have not been molecularly defined. In THP-1 cells with restricted HIV expression, there is an absence of DNA-protein binding complex formation with the HIV-1 promoter-enhancer associated with markedly less viral RNA production. This absence of binding was localized to the NF-kappa B region of the HIV-1 enhancer; the 65-kDa plus 50-kDa NF-kappa B heterodimer was preferentially lost. Adding purified NF-kappa B protein to nuclear extracts from cells with restricted expression overcomes this lack of binding. In addition, treatment of these nuclear extracts with sodium deoxycholate restored their ability to form the heterodimer, suggesting the presence of an inhibitor of NF-kappa B activity. Furthermore, treatment of nuclear extracts from these cells that had restricted expression with lipopolysaccharide increased viral production and NF-kappa B activity. Antiserum specific for NF-kappa B binding proteins, but not c-rel-specific antiserum, disrupted heterodimer complex formation. Thus, both NF-kappa B-binding complexes are needed for optimal viral transcription. Binding of the 65-kDa plus 50-kDa heterodimer to the HIV-1 enhancer can be negatively regulated in monocytes, providing one mechanism restricting HIV-1 gene expression.

MEDLINE:91277630

**Human tumor necrosis factor alpha gene regulation in phorbol ester stimulated T and B cell lines.**

The minimal region of the human tumor necrosis factor alpha (TNF-alpha) gene promoter necessary for its transcriptional induction by phorbol esters (PMA) in human T and B lymphocyte cell lines has been localized between -52 and +89 nucleotides (nt) relative to the gene's transcriptional start site. Comparison of these sequences to those required to mediate virus or lipopolysaccharide (LPS) induction of the gene reveal significant differences, and thus, the sequence requirements for PMA induction are distinct from those that mediate induction by virus or LPS. Although three sites in the TNF-alpha promoter (kappa 1, kappa 2, and kappa 3) specifically bind the transcription factor NF-kappa B in lymphoid nuclear extracts, TNF-alpha mRNA induction by PMA does not correlate with NF-kappa B binding activities displayed by different T and B cell lines. Moreover, kappa 1-kappa 3 can each be deleted from the TNF-alpha promoter with little effect on the gene's inducibility by PMA. Therefore, TNF-alpha mRNA induction by PMA, like its induction by virus and LPS, is not primarily mediated by NF-kappa B, but rather is mediated through other sequences and protein factors. Surprisingly, multimers of kappa 1-kappa 3 can confer PMA inducibility on a heterologous promoter in a B (Raji), but not a T (HUT78) cell line. However they are not functional on a truncated TNF-alpha promoter, indicating that promoter context and cell type specificity influence the PMA inducible function of these NF-kappa B binding sites.

MEDLINE:92068245

**Constitutive activation of NF-kB in human thymocytes.**

NF-kB is a eukaryotic transcription regulatory factor. In T cells and T cell lines, NF-kB is bound to a cytoplasmic proteic inhibitor, the IkB. Treatment of T cells with mitogens (phorbol esters) or cytokines (TNF alpha) induces NF-kB nuclear translocation and the subsequent expression of NF-kB dependent T cell genes. Here we examined the activation of NF-kB in human T cell thymic progenitors. We report differences in (Ca2+)i requirement for NF-kB activation in thymocytes as compared to mature T cells. Furthermore, our results indicated that thymocytes have a constitutively active form of NF-kB, suggesting that they are activated in vivo.

MEDLINE:92179751

**[Regulatory effect of insulin on glucocorticoid receptor in human peripheral leukocytes]**

The regulatory effect of insulin on the specific binding power of glucocorticoid receptor (GR) of human leukocytes was assessed by the unoccupied receptor sites capable of combining with [3H] labelled dexamethasone measured at 3 and 24 h after incubation with various concentrations of insulin added to the medium. After 3 h incubation the specific binding power with [3H] Dex was decreased by 23.3 +/- 10.0, 32.2 +/- 13.2 and 54.3 +/- 9.2% (P greater than 0.05, P greater than 0.05 and P less than 0.01 as compared with the control value of 100 in the absence of insulin) respectively in the presence of 20 mU/L (physiological testing concentration), 200 mU/L (physiological upper limit) and 2,000 mU/L (pharmacological concentration) insulin in the incubation medium. After 24 h incubation the decrease of these values increased respectively to 43.5 +/- 19.0, 56.1 +/- 20.7 and 80.2 +/- 15.5 (P less than 0.05, P less than 0.01 and P less than 0.01 compared with control). Thus the inhibitory effect of insulin on the GR binding power is both dose- and time-dependent, which strongly suggests that GR is tonically controlled by insulin concentration change under physiological conditions.

MEDLINE:91170801

**Kappa B binding proteins are constitutively expressed in an IL-2 autocrine human T cell line.**

The IL-2 and the IL-2-R alpha genes are both expressed transiently in normal T lymphocytes after Ag or mitogen activation. In contrast, the human T cell line, IARC 301, expresses these two genes constitutively and we have previously demonstrated that its growth depends on the autocrine production of this T cell growth factor and high affinity IL-2R. To dissect the molecular basis for the unusual persistent expression of the IL-2 and IL-2-R alpha genes in these IARC 301 T cells, we have analyzed the interactions of constitutively expressed nuclear proteins with the 5' flanking regions of the IL-2 and IL-2-R alpha genes using both DNase I footprinting and gel retardation techniques. We have found that a region in both genes (-276 to -250 for IL-2-R alpha and -203 to -183 for IL-2), which corresponds to a kappa B enhancer element, is specifically protected by nuclear proteins from IARC 301. In agreement with this finding, both the IL-2 and IL-2-R alpha promoters are active in transient transfection assays in IARC 301 cells. In contrast, mutation of the kappa B enhancer results in markedly attenuated activities of both promoters. Two proteins binding the kappa B sequence, NF-kappa B and KBF1, are constitutively expressed in IARC 301 nuclei and induced by PMA and PHA in Jurkat. They bind to the kappa B motifs with different relative affinities that may reflect their different contribution in the expression of various promoters.

MEDLINE:92118719

**Kappa B-specific DNA binding proteins are differentially inhibited by enhancer mutations and biological oxidation.**

Kappa B (kappa B) enhancer binding proteins isolated from the nuclei of activated human T cells produce two distinct nucleoprotein complexes when incubated with the kappa B element from the interleukin-2 receptor-alpha (IL-2R alpha) gene. These two DNA-protein complexes are composed of at least four host proteins (p50, p55, p75, p85), each of which shares structural similarity with the v-rel oncogene product. Nuclear expression of these proteins is induced with distinctly biphasic kinetics following phorbol ester activation of T cells (p55/p75 early and p50/p85 late). DNA-protein crosslinking studies have revealed that the more rapidly migrating B2 complex contains both p50 and p55 while the more slowly migrating B1 complex is composed of p50, p55, p75, and p85. Site-directed mutagenesis of the wild-type IL-2R alpha kappa B enhancer (GGGGAATCTCCC) has revealed that the binding of p50 and p55 (B2 complex) is particularly sensitive to alteration of the 5' triplet of deoxyguanosine residues. In contrast, formation of the B1 complex, reflecting the binding of p75 and p85, critically depends upon the more 3' sequences of this enhancer element. DNA binding by all four of these Rel-related factors is blocked by selective chemical modification of lysine and arginine residues, suggesting that both of these basic amino acids are required for binding to the kappa B element. Similarly, covalent modification of free sulfhydryl groups with diamide (reversible) or N-ethylmaleimide (irreversible) results in a complete loss of DNA binding activity. In contrast, mild oxidation with glucose oxidase selectively inhibits p75 and p85 binding while not blocking p50 and p55 interactions. These findings suggest that reduced cysteine thiols play an important role in the DNA binding activity of this family of Rel-related transcription factors.

MEDLINE:92052243

**cAMP-dependent regulation of proenkephalin by JunD and JunB: positive and negative effects of AP-1 proteins.**

We demonstrate that JunD, a component of the AP-1 transcription factor complex, activates transcription of the human proenkephalin gene in a fashion that is completely dependent upon the cAMP-dependent protein kinase, protein kinase A. Activation of proenkephalin transcription by JunD is dependent upon a previously characterized cAMP-, phorbol ester-, and Ca(2+)-inducible enhancer, and JunD is shown to bind the enhancer as a homodimer. Another component of the AP-1 transcription complex, JunB, is shown to inhibit activation mediated by JunD. As a homodimer JunB is unable to bind the enhancer; however in the presence of c-Fos, high-affinity binding is observed. Furthermore, JunD is shown to activate transcription of genes linked to both cAMP and phorbol ester response elements in a protein kinase A-dependent fashion, further blurring the distinction between these response elements. These results demonstrate that the transcriptional activity of an AP-1-related protein is regulated by the cAMP-dependent second-messenger pathway and suggest that JunD and other AP-1-related proteins may play an important role in the regulation of gene expression by cAMP-dependent intracellular signaling pathways.

MEDLINE:92135145

**Activity of the kappa B enhancer of the interleukin-2 receptor alpha chain in somatic cell hybrids is accompanied by the nuclear localization of NF-kappa B.**

The two nuclear proteins NF-kappa B (consisting of subunits p50 an dp65) and the DNA-binding subunit of NF-kappa B (p50) by itself, also called KBF1, are constitutively expressed and localized in the nucleus of the human T-cell line IARC 301.5. In order to define the roles of these two factors, which bind to the same kappa B enhancers, in transcription activation we have prepared somatic cell hybrids between IARC 301.5 and a murine myeloma. Most hybrids express both KBF1 and NF-kappa B in their nuclei, but one hybrid expresses only KBF1. The kappa B enhancer of the gene encoding the interleukin-2 (IL-2) receptor alpha chain (IL-2R alpha) is functional only in the hybrids expressing nuclear NF-kappa B. These findings show that nuclear NF-kappa B is necessary to activate the kappa B enhancer, while KBF1 by itself is not sufficient. We propose that KBF1 is a competitive inhibitor of NF-kappa B and discuss how these factors may be involved in the transient expression of IL-2 and IL-2 alpha genes during the immune response.

MEDLINE:91355651

**NF-kappa B activation by tumor necrosis factor alpha in the Jurkat T cell line is independent of protein kinase A, protein kinase C, and Ca(2+)-regulated kinases.**

NF-kappa B is a DNA-binding regulatory factor able to control transcription of a number of genes, including human immunodeficiency virus (HIV) genes. In T cells, NF-kappa B is activated upon cellular treatment by phorbol esters and the cytokine tumor necrosis factor alpha (TNF alpha). In the present work, we investigated the molecular events leading to NF-kappa B activation by TNF alpha in a human T cell line (Jurkat) and its subclone JCT6, which presents a deficiency in the PKA transduction pathway. We found that in both cell lines, both phorbol ester and TNF alpha were able to activate NF-kappa B. Phorbol activation was positively modulated by Ca2+ influx while TNF alpha activation was not. Furthermore, while PMA activation was inhibited by the PKC inhibitor staurosporin, the TNF alpha effect was unchanged. TNF alpha did not activate cAMP production and its signal was not modulated by cAMP activators. Moreover, cAMP activators did not activate NF-kappa B in Jurkat cells. Thus, TNF alpha-induced NF-kappa B activation was found to be mediated by none of the major signal-mediating kinases such as protein kinase C (PKC), protein kinase A, or Ca(2+)-regulated kinases. Furthermore, we found that cytoplasmic acidification facilitated NF-kappa B activation by both TNF alpha and PKC, by a mechanism that increases NF-kappa B/I kappa B dissociation without affecting the NF-kappa B translocation step.

MEDLINE:91172211

**The functional domains of the murine Thy-1 gene promoter.**

The Thy-1 gene promoter resembles a "housekeeping" promoter in that it is located within a methylation-free island, lacks a canonical TATA box, and displays heterogeneity in the 5'-end termini of the mRNA. Using transgenic mice, we show that this promoter does not confer any tissue specificity and is active only in a position-dependent manner. It can only be activated in a tissue-specific manner by elements that lie downstream of the initiation site. We have analyzed the functional domains of the minimal Thy-1 promoter and show that the dominant promoter elements consist of multiple binding sites for the transcription factor Sp1, an inverted CCAAT box, and sequences proximal to the transcription start site. DNase I and gel mobility shift assays show the binding of a number of nuclear factors to these elements, including Sp1 and CP1. Our results show that the structure of this promoter only permits productive interactions of the two transcription factors Sp1 and CP1 with the basal transcription machinery in the presence of enhancer sequences.

MEDLINE:91117203

**Nuclear factor kappa B activates proenkephalin transcription in T lymphocytes.**

Upon activation, T lymphocytes accumulate high levels of the neuropeptide enkephalin which correlate with high levels of proenkephalin mRNA in the cells. Here we investigated the transcriptional basis for these changes. The proenkephalin promoter contains a sequence GGGGACGTCCCC, named B2, which is similar to the kappa B sequence GGGGACTTTCC, the binding site of the transcription factor nuclear factor (NF)-kappa B. Activation of T lymphocytes induces an NF-kappa B-like binding activity to the B2 site, concomitant with activation of the proenkephalin promoter. Mutations at the B2 site abolish this transcriptional activation. The purified homodimer (two p50s) of the DNA-binding subunit of NF-kappa B binds the B2 site of proenkephalin relatively better than does the heterotetramer (two p65s plus two p50s) form of the factor. Thus, it appears that the T-cell-specific activation of the proenkephalin promoter is mediated by NF-kappa B. However, as NF-kappa B is ubiquitous and the transcriptional activation through the B2 site is T cell specific, yet another T-cell-specific factor which synergizes with NF-kappa B should be considered.

MEDLINE:90287178

**Involvement of cyclic AMP-dependent protein kinases in the signal transduction pathway for interleukin-1.**

Expression of a highly specific protein inhibitor for cyclic AMP-dependent protein kinases in interleukin-1 (IL-1)-responsive cells blocked IL-1-induced gene transcription that was driven by the kappa immunoglobulin enhancer or the human immunodeficiency virus long terminal repeat. This inhibitor did not affect protein kinase C-mediated gene transcription, suggesting that cyclic AMP-dependent protein kinases are involved in the signal transduction pathway for IL-1 in a number of responsive cell types.

MEDLINE:91045897

**Stimulation of a human T-cell clone with anti-CD3 or tumor necrosis factor induces NF-kappa B translocation but not human immunodeficiency virus 1 enhancer-dependent transcription.**

The expression of transiently transfected expression vectors under the control of the long terminal repeat (LTR) of the human immunodeficiency virus (HIV) or its enhancer sequence and the translocation of the HIV enhancer-binding protein NF-kappa B were analyzed in two human T-cell clones stimulated through their T-cell receptor complex or by tumor necrosis factor or phorbol 12-myristate 13-acetate. We found a dissociation of NF-kappa B translocation from transactivation of either the HIV LTR or the HIV enhancer. Interleukin 2 induced proliferation but not NF-kappa B translocation or LTR transactivation. Phorbol ester or specific antigen recognition induced HIV LTR transactivation, whereas stimulation with tumor necrosis factor or antibody to CD3 did not. The two latter signals were nevertheless able to induce NF-kappa B translocation with a pattern in the band-shift assay indistinguishable from that observed using phorbol ester. Our finding that induction of NF-kappa B by tumor necrosis factor or antibody to CD3 is not sufficient to induce HIV enhancer-dependent transcription in cloned T cells contrasts with results obtained in most lymphoblastoid T-cell lines and indicates that normal T lymphocytes differ from tumoral T cells in terms of requirements for HIV LTR activation. Furthermore, our results suggest that events linked to T-cell activation, in addition to NF-kappa B translocation per se, induce functional interactions of the NF-kappa B complex with the HIV enhancer.

MEDLINE:90337320

**A novel B-cell lineage-specific transcription factor present at early but not late stages of differentiation.**

A novel B-cell-specific transcription factor, BSAP, was identified as a mammalian homolog of the sea urchin protein TSAP, which interacts with the promoters of four tissue-specific late histone H2A-2 and H2B-2 genes. As shown by mobility-shift, methylation interference, and mutational analyses, the mammalian protein BSAP recognizes all four sea urchin binding sites in a manner indistinguishable from TSAP; however, the two proteins differ in molecular weight. BSAP is exclusively restricted to the B-cell lineage of lymphoid differentiation. Its expression appears to be activated during pro-B-cell development, is abundant at the pre-B- and mature B-cell stages, but is absent in terminally differentiated plasma cells. Moreover, BSAP is clearly a B-cell-specific transcription factor, as a wild-type but not a mutant TSAP-binding site of the sea urchin functions only in transfected B cells as an upstream promoter element. Competition experiments did not reveal any high-affinity binding site for BSAP in known regulatory regions of immunoglobulin and class II major histocompatibility (MHC) genes, suggesting that BSAP is a regulator of a different set of B-lymphoid-specific genes.

MEDLINE:90317911

**Inducible nuclear factor binding to the kappa B elements of the human immunodeficiency virus enhancer in T cells can be blocked by cyclosporin A in a signal-dependent manner.**

Cyclosporin A (CsA) is thought to exert its immunosuppressive effects by inhibiting the expression of a distinct set of lymphokine genes which are induced upon T-cell activation, among them the gene coding for interleukin-2. In addition, the activation of the human immunodeficiency virus (HIV) is partially suppressed. To better understand the molecular mechanisms underlying suppression by CsA, we have investigated the effects of this drug on transcription factors in T cells. Here we report that the formation of two distinct mitogen-inducible DNA-binding complexes, the kappa B complex within the HIV enhancer and the NFAT-1 complex within the interleukin-2 enhancer, is inhibited in the presence of CsA. The kappa B-binding activity with the HIV enhancer is inhibited only if it is activated via the mitogen phytohemagglutinin whereas phorbol myristate acetate-mediated activation is completely insensitive to the drug. This suggests a model in which functionally indistinguishable kappa B complexes can be activated via two separate pathways of signal transduction distinguishable by CsA.

MEDLINE:91190842

**Purification of TCF-1 alpha, a T-cell-specific transcription factor that activates the T-cell receptor C alpha gene enhancer in a context-dependent manner.**

The differentiation of T cells into functionally diverse subpopulations is controlled in part, by transcriptional activation and silencing; however, little is known in detail about the proteins that influence this developmental process. We have purified a new T-cell-specific factor, TCF-1 alpha, that is implicated in the activation of genes encoding a major component of the human T-cell receptor (TCR). TCF-1 alpha, originally identified and purified through its binding sites on the HIV-1 promoter, was found to bind to the TCR alpha enhancer and to promoters for several genes expressed at significantly earlier stages of T-cell development than the TCR alpha gene (e.g., p56lck and CD3 delta). Sequences related to the TCF-1 alpha binding motif (5'-GGCACCCTTTGA-3') are also found in the human TCR delta (and possibly TCR beta) enhancers. Southwestern and gel renaturation experiments with the use of purified protein fractions revealed that TCF-1 alpha activity is derived from a family of 57- to 53-kD proteins that are abundantly expressed in mature and immature T-cell lines (Jurkat, CCRF-CEM) and not in mature B cells (JY, Namalwa) or nonlymphoid (HeLa) cell lines. A small 95-bp fragment of the TCR alpha control region that contains the TCF-1 alpha binding site juxtaposed between a cAMP-response element (the CRE or T alpha 1 motif) and the binding site for a distinct lymphoid-specific protein (TCF-2 alpha) behaved as a potent T-cell-specific enhancer in vivo. Tandem copies of this enhancer functioned synergistically in mature (Jurkat) T-cell lines as well as resting and activated immature (CCRF-CEM) T-cell lines. Mutation of the TCF-1 alpha binding site diminished enhancer activity and disrupted the synergism observed in vivo between tandem enhancer repeats. The TCF-1 alpha binding site was also required for TCR alpha enhancer activity in transcriptionally active extracts from Jurkat but not HeLa cells, confirming that TCF-1 alpha is a T-cell-specific transcription factor. Curiously, the TCF-1 alpha binding element was inactive in vivo when removed from its neighboring elements on the TCR alpha enhancer and positioned in one or more copies upstream of a heterologous promoter. Thus, the transcriptional activity of TCF-1 alpha appears to depend on the TCF-2 alpha and T alpha 1 (CREB) transcription factors and the context of its binding site within the TCR alpha enhancer.

MEDLINE:90346297

**Tandem AP-1-binding sites within the human beta-globin dominant control region function as an inducible enhancer in erythroid cells.**

A powerful enhancer has been mapped to an 18-bp DNA segment located 11 kb 5' to the human epsilon-globin gene within the dominant control or locus-activating region. This enhancer is inducible in K562 human erythroleukemia cells, increasing linked gamma-globin promoter/luciferase gene expression to 170-fold over an enhancerless construct. The enhancer consists of tandem AP-1-binding sites, phased 10 bp apart, which are both required for full activity. DNA-protein binding assays with nuclear extracts from induced cells demonstrate a high molecular weight complex on the enhancer. The formation of this complex also requires both AP-1 sites and correlates with maximal enhancer activity. Induction of the enhancer may have a role in the increase in globin gene transcription that characterizes erythroid maturation. Enhancer activity appears to be mediated by the binding of a complex of proteins from the jun and fos families to tandem AP-1 consensus sequences.

MEDLINE:90220600

**Identification of a novel factor that interacts with an immunoglobulin heavy-chain promoter and stimulates transcription in conjunction with the lymphoid cell-specific factor OTF2.**

The tissue-specific expression of the MOPC 141 immunoglobulin heavy-chain gene was studied by using in vitro transcription. B-cell-specific transcription of this gene was dependent on the octamer element 5'-ATGCAAAG-3', located in the upstream region of this promoter and in the promoters of all other immunoglobulin heavy- and light-chain genes. The interaction of purified octamer transcription factors 1 and 2 (OTF1 and OTF2) with the MOPC 141 promoter was studied by using electrophoretic mobility shift assays and DNase I footprinting. Purified OTF1 from HeLa cells and OTF1 and OTF2 from B cells bound to identical sequences within the heavy-chain promoter. The OTF interactions we observed extended over the heptamer element 5'-CTCAGGA-3', and it seems likely that the binding of the purified factors involves cooperation between octamer and heptamer sites in this promoter. In addition to these elements, we identified a second regulatory element, the N element with the sequence 5'-GGAACCTCCCCC-3'. The N element could independently mediate low levels of transcription in both B-cell and HeLa-cell extracts, and, in conjunction with the octamer element, it can promote high levels of transcription in B-cell extracts. The N element bound a transcription factor, NTF, that is ubiquitous in cell-type distribution, and NTF was distinct from any of the previously described proteins that bind to similar sequences. Based on these results, we propose that NTF and OTF2 interactions (both with their cognate DNA elements and possibly at the protein-protein level) may be critical to B-cell-specific expression and that these interactions provide additional pathways for regulating gene expression.

MEDLINE:90158596

**NF-kappa B as inducible transcriptional activator of the granulocyte-macrophage colony-stimulating factor gene.**

The expression of the gene encoding the granulocyte-macrophage colony-stimulating factor (GM-CSF) is induced upon activation of T cells with phytohemagglutinin and active phorbolester and upon expression of tax1, a transactivating protein of the human T-cell leukemia virus type I. The same agents induce transcription from the interleukin-2 receptor alpha-chain and interleukin-2 genes, depending on promoter elements that bind the inducible transcription factor NF-kappa B (or an NF-kappa B-like factor). We therefore tested the possibility that the GM-CSF gene is also regulated by a cognate motif for the NF-kappa B transcription factor. A recent functional analysis by Miyatake et al. (S. Miyatake, M. Seiki, M. Yoshida, and K. Arai, Mol. Cell. Biol. 8:5581-5587, 1988) described a short promoter region in the GM-CSF gene that conferred strong inducibility by T-cell-activating signals and tax1, but no NF-kappa B-binding motifs were identified. Using electrophoretic mobility shift assays, we showed binding of purified human NF-kappa B and of the NF-kappa B activated in Jurkat T cells to an oligonucleotide comprising the GM-CSF promoter element responsible for mediating responsiveness to T-cell-activating signals and tax1. As shown by a methylation interference analysis and oligonucleotide competition experiments, purified NF-kappa B binds at positions -82 to -91 (GGGAACTACC) of the GM-CSF promoter sequence with an affinity similar to that with which it binds to the biologically functional kappa B motif in the beta interferon promoter (GGGAAATTCC). Two kappa B-like motifs at positions -98 to -108 of the GM-CSF promoter were also recognized but with much lower affinities. Our data provide strong evidence that the expression of the GM-CSF gene following T-cell activation is controlled by binding of the NF-kappa B transcription factor to a high-affinity binding site in the GM-CSF promoter.

MEDLINE:90317873

**The Epstein-Barr virus (EBV) BMRF1 promoter for early antigen (EA-D) is regulated by the EBV transactivators, BRLF1 and BZLF1, in a cell-specific manner.**

The Epstein-Barr virus early antigen diffuse component (EA-D) is essential for Epstein-Barr virus DNA polymerase activity, and its activity is suppressed during latent infection. We investigated the regulation of the promoter (BMRF1) for this early gene by studying its responsiveness in vitro to two immediate-early viral transactivators, BZLF1 (Z) and BRLF1 (R), focusing on the differences in response in lymphoid cells and epithelial cells. In lymphoid cells, Z or R alone produced only small increases in EA-D promoter activity, whereas both transactivators together produced a large stimulatory effect. In epithelial cells, the Z transactivator alone produced maximal stimulation of the EA-D promoter; the effect of R and Z together was no greater than that of Z alone. Deletional analysis and site-directed mutagenesis of the EA-D promoter demonstrated that in epithelial cells the potential AP-1 binding site plays an essential role in Z responsiveness, although sequences further upstream are also important. In lymphoid cells, only the upstream sequences are required for transactivation by the Z/R combination, and the AP-1 site is dispensable. These data suggest that EA-D (BMRF1) promoter regulation by Z and R is cell type specific and appears to involve different mechanisms in each cell type.

MEDLINE:91079583

**Activation of human CD4 T lymphocytes. Interaction of fibronectin with VLA-5 receptor on CD4 cells induces the AP-1 transcription factor.**

Fibronectin synergized with anti-CD3 antibody to promote CD4 cell proliferation in a serum-free culture system whereas no proliferation was observed when CD4 cells were cultured with anti-CD3 alone or fibronectin alone. In addition, anti-CD29 (integrin beta 1) as well as anti-VLA-5 (human fibronectin receptor) antibodies blocked this CD4 cell activation in this system. Although anti-CD3 alone or fibronectin alone cannot induce IL-2 message by CD4 cells, the combination of anti-CD3 plus fibronectin induced IL-2 message by CD4 cells. In an analysis of the molecular mechanism by which IL-2 message was generated, we showed that a fibronectin-VLA-5 fibronectin receptor interaction may contribute an independent signal distinct from the CD3 pathway of activation by the induction of an AP-1 transcriptional factor. Thus the VLA-5 fibronectin receptor on CD4 cells can play a complementary role in CD3-TCR-mediated signal transduction through its interaction with fibronectin.

MEDLINE:90140708

**Two distinct transcription factors that bind the immunoglobulin enhancer microE5/kappa 2 motif.**

Activity of the immunoglobulin heavy and kappa light chain gene enhancers depends on a complex interplay of ubiquitous and developmentally regulated proteins. Two complementary DNAs were isolated that encode proteins, denoted ITF-1 and ITF-2, that are expressed in a variety of cell types and bind the microE5/kappa 2 motif found in both heavy and kappa light chain enhancers. The complementary DNAs are the products of distinct genes, yet both ITF-1 and ITF-2 are structurally and functionally similar. The two proteins interact with one another through their putative helix-loop-helix motifs and each possesses a distinct domain that dictates transcription activation.

MEDLINE:91092267

**The actions of cyclosporin A and FK506 suggest a novel step in the activation of T lymphocytes.**

Cyclosporin A and FK506 are immunosuppressive compounds that have similar inhibitory effects on the expression of several lymphokines produced by T lymphocytes. Despite their similar effects the drugs bind to two different cytosolic protein, cyclophilin and FKBP respectively, which raises the possibility that they have different modes of action. Using constructs in which mRNA production controlled by a specific transcription factor could be readily measured we found that both cyclosporin A and FK506 completely inhibited transcription activated by NF-AT, NFIL2 A, NFIL2 B and partially inhibited transcription activated by NF kappa B. Cyclosporin A and FK506 inhibited only transcriptional activation that was dependent on Ca2+ mobilization. However, cyclosporin A and FK506 did not inhibit Ca2+ mobilization dependent expression of c-fos mRNA indicating that only a subset of signalling pathways regulated by Ca2+ is sensitive to these drugs. Furthermore, we did not observe any qualitative differences between the effect of cyclosporin A and FK506 on six different transcription factors which suggests that these drugs may interfere with the activity of a novel Ca2+ dependent step that regulates several transcription factors.

MEDLINE:90147621

**Characterization of the human immunodeficiency virus type 1 enhancer-binding proteins from the human T-cell line Jurkat.**

The transcription of the human immunodeficiency virus type 1 (HIV-1) is under the control of cellular proteins that bind to the viral long terminal repeat (LTR). Among the protein-binding regions of the HIV-1 LTR is the transcription-enhancer region. We show that at least one inducible, C1, and one constitutive, C2, protein can bind to the HIV enhancer in Jurkat cells. The two proteins differ in their surface charge, since they are separable by anion-exchange chromatography. Bivalent cations such as Mg2+ and Zn2+ differentially affect their binding to oligonucleotides which contain the HIV-enhancer domain. Both C1 and C2 proteins also bind to a similar sequence found in the interleukin-2-receptor alpha-subunit enhancer. The inducible C1 protein was partially purified by three chromatographic steps and characterized by u.v. cross-linking as a 47 kDa protein.

MEDLINE:90279052

**A novel T-cell protein which recognizes a palindromic sequence in the negative regulatory element of the human immunodeficiency virus long terminal repeat.**

Two major protein-binding sites within the negative regulatory element of the human immunodeficiency virus type 1 long terminal repeat have been identified. One (site B) contained a palindromic sequence with homology to steroid/thyroid hormone response elements but was distinct from previously described binding sites of this class. A novel T-cell protein recognized the palindromic sequence within site B and also bound estrogen- or thyroid hormone-response elements with lower affinity. A 7-base-pair mutation in the site B palindrome, which destroyed protein binding, resulted in increased expression from the human immunodeficiency virus type 1 long terminal repeat in T cells.

MEDLINE:90335211

**The 56-59-kilodalton protein identified in untransformed steroid receptor complexes is a unique protein that exists in cytosol in a complex with both the 70- and 90-kilodalton heat shock proteins.**

It has previously been shown that 9S, untransformed progestin, estrogen, androgen, and glucocorticoid receptor complexes in rabbit uterine and liver cytosols contain a 59-kDa protein [Tai, P.K., Maeda, Y., Nakao, K., Wakim, N.G., Duhring, J.L., & Faber, L.E.(1986) Biochemistry 25, 5269-5275]. In this work we show that the monoclonal antibody KN 382/EC1 raised against the rabbit 59-kDa protein reacts with 9S, untransformed glucocorticoid receptor complexes in cytosol prepared from human IM-9 lymphocytes but not with 4S salt-transformed receptors. The human protein recognized by the EC1 antibody is a 56-kDa protein (p56) of moderate abundance located predominantly in the cytoplasm by indirect immunofluorescence. There are at least six isomorphs of p56 by two-dimensional gel analysis. N-Terminal sequencing (20 amino acids) shows that p56 is a unique human protein. When p56 is immunoadsorbed from IM-9 cell cytosol, both the 70- and 90-kDa heat shock proteins are coadsorbed in an immune-specific manner. Neither heat shock protein reacts directly with the EC1 antibody. We conclude that p56 exists in cytosol in a higher order complex containing hsp70 and hsp90, both of which in turn have been found to be associated with untransformed steroid receptors.

MEDLINE:98038598

**Ras-related GTP-binding proteins and leukocyte signal transduction.**

Many aspects of leukocyte function are regulated by both heterotrimeric and Ras-related GTP-binding proteins, but there is little definite information about their roles in the specialized processes utilized by leukocytes for cell killing. Recent progress in understanding the regulation of the phagocyte NADPH oxidase by the Rac GTP-binding proteins provides a basis for defining the operational characteristics of one such phagocyte system. It is clear from various studies that the activity of the NADPH oxidase can be modulated through the regulation of the GTP-GDP state of Rac. Proteins exist in leukocytes able to modify GTP-binding protein function in this manner, and their activity may be regulated by signals generated on phagocyte stimulation. Proteins of the Ras superfamily are likely to be involved in a variety of normal phagocyte functions through their ability to modulate the assembly of actin filaments, direct vesicle trafficking and fusion, and so forth.

MEDLINE:96068726

**The myeloid zinc finger gene, MZF-1, regulates the CD34 promoter in vitro.**

MZF-1 is a C2H2 zinc finger gene encoding a putative transcriptional regulator of myeloid differentiation. The MZF-1 protein contains 13 C2H2 zinc fingers arranged in bipartite DNA binding domains containing zinc fingers through 4 and, in the carboxy-terminus, 5 through 13. We previously identified the DNA consensus binding site recognized by the two DNA binding domains. To assess the transcription regulatory function of MZF-1, the full-length MZF-1 coding region was fused to the DNA binding domain of the yeast transactivator GAL4. The expression vector was cotransfected with the chloramphenicol acetyl transferase (CAT) reporter gene regulated by the thymidine kinase promoter containing GAL4 DNA binding sites into NIH 3T3, 293, K562, and Jurkat cell lines. MZF-1 represses CAT reporter gene expression via GAL4 binding sites in the nonhematopoietic cell lines NIH 3T3 and 293. In contrast, MZF-1 activates CAT reporter gene expression in the hematopoietic cell lines K562 and Jurkat. The MZF-1 binding sites are present in the promoters of several genes expressed during myeloid differentiation, including the CD34 promoter. MZF-1 transcriptional regulation of this physiologically relevant promoter was assessed in both hematopoietic and nonhematopoietic cell lines. Recombinant MZF-1 protein specifically binds to the consensus binding sites in the CD34 promoter in mobility shift assays. MZF-1 expression vectors were cotransfected with the luciferase reporter plasmids regulated by the CD34 promoter into both nonhematopoietic and hematopoietic cell lines. As with the heterologous DNA binding domain, MZF-1 represses reporter gene expression in nonhematopoietic cell lines and activates expression in hematopoietic cell lines. Activation of CD34 expression in hematopoietic cell lines is dependent on the presence of intact MZF-1 binding sites. The cell type-specific regulation of the CD34 promoter by MZF-1 suggests the presence of tissue-specific regulators/adapters or differential MZF-1 modifications that determine MZF-1 transcriptional regulatory function.

MEDLINE:95378799

**Activation and expression of the nuclear factors of activated T cells, NFATp and NFATc, in human natural killer cells: regulation upon CD16 ligand binding.**

The putative factors that couple the signal transduction from surface receptors to the activation of cytokine synthesis in natural killer (NK) cells have not been elucidated. We report here that the nuclear factor of activated T cells (NFATp), a cyclosporin A (CsA)-sensitive factor that regulates the transcription of several cytokines, mediates CD16-induced activation of cytokine genes in human NK cells. CD16 (Fc gamma RIIIA)-induced expression of cytokine mRNA in NK cells occurs via a CsA-sensitive and Ca(2+)-dependent mechanism. Stimulation of NK cells with CD16 ligands induces NFAT-like DNA binding activity in the nuclear extracts from these cells, as detected in electrophoretic mobility shift assays. This occurs with fast kinetics after stimulation, via a CsA-sensitive and Ca(2+)-dependent mechanism that does not require de novo protein synthesis. NK cell NFAT is present in the cytosol of nonstimulated cells, migrates to the nucleus upon stimulation, and can associate with AP-1. Two distinct molecules, NFATp and NFATc, have been reported to mediate NFAT activity. The results of supershift assays using NFATp- and NFATc- specific antibodies indicate that NK cell activation early after CD16 ligand binding involves primarily, if not exclusively, NFATp, and Western blot analysis shows that this has the same electrophoretic mobility (approximately 120 kD) as that of T lymphocytes. NK cells do not express NFATc constitutively, but NFATc mRNA accumulation is induced in these cells within 2 h of stimulation with CD16 ligands. However, supershift assays using the available mAb recognizing the T cell NFATc revealed no detectable NFATc protein in nuclear and cytoplasmic extracts from CD16- or phorbol ester-stimulated cells at any time tested, up to 4 h. These results provide the first direct evidence that both CsA-sensitive transcription factors, NFATp and NFATc, are expressed in human NK cells, and that their activation and/or expression can be regulated in primary cells by a single stimulus, that, in the case of CD16 in NK cells, results in early activation of NFATp and subsequently induced expression of NFATc mRNA.

MEDLINE:95372363

**Interleukin 2 signaling involves the phosphorylation of Stat proteins.**

One of the most important cytokines involved in immune response regulation is interleukin 2 (IL-2), a potent activator of the proliferation and function of T lymphocytes and natural killer cells. The mechanisms by which the effects of IL-2 are propagated within cells are not understood. While the binding of IL-2 to its receptor was recently shown to lead to the activation of two kinases, Jak-1 and Jak-3, subsequent steps in the signaling pathway to the nucleus that lead to the activation of specific genes had not been characterized. Since many cytokines that activate Jak kinases also lead to the tyrosine phosphorylation and activation of members of the Stat family of transcription factors, the ability of IL-2 to trigger Stat phosphorylation was examined. Exposure of activated human T lymphocytes or of a natural killer cell line (NKL) to IL-2 leads to the phosphorylation of Stat1 alpha, Stat1 beta, and Stat3, as well as of two Stat-related proteins, p94 and p95. p94 and p95 share homology with Stat1 at the phosphorylation site and in the Src homology 2 (SH2) domain, but otherwise are immunologically distinct from Stat1. These Stat proteins were found to translocate to the nucleus and to bind to a specific DNA sequence. These findings suggest a mechanism by which IL-2 binding to its receptor may activate specific genes involved in immune cell function.

MEDLINE:96074633

**Staphylococcal enterotoxins modulate interleukin 2 receptor expression and ligand-induced tyrosine phosphorylation of the Janus protein-tyrosine kinase 3 (Jak3) and signal transducers and activators of transcription (Stat proteins).**

Staphylococcal enterotoxins (SE) stimulate T cells expressing the appropriate variable region beta chain of (V beta) T-cell receptors and have been implicated in the pathogenesis of several autoimmune diseases. Depending on costimulatory signals, SE induce either proliferation or anergy in T cells. In addition, SE can induce an interleukin-2 (IL-2) nonresponsive state and apoptosis. Here, we show that SE induce dynamic changes in the expression of and signal transduction through the IL-2 receptor (IL-2R) beta and gamma chains (IL-2R beta and IL-2R gamma) in human antigen-specific CD4+ T-cell lines. Thus, after 4 hr of exposure to SEA and SEB, the expression of IL-2R beta was down-regulated, IL-2R gamma was slightly up-regulated, while IL-2R alpha remained largely unaffected. The changes in the composition of IL-2Rs were accompanied by inhibition of IL-2-induced tyrosine phosphorylation of the Janus protein-tyrosine kinase 3 (Jak3) and signal transducers and activators of transcription called Stat3 and Stat5. In parallel experiments, IL-2-driven proliferation was inhibited significantly. After 16 hr of exposure to SE, the expression of IL-2R beta remained low, while that of IL2R alpha and IL2R gamma was further up-regulated, and ligand-induced tyrosine phosphorylation of Jak3 and Stat proteins was partly normalized. Yet, IL-2-driven proliferation remained profoundly inhibited, suggesting that signaling events other than Jak3/Stat activation had also been changed following SE stimulation. In conclusion, our data suggest that SE can modulate IL-2R expression and signal transduction involving the Jak/Stat pathway in CD4+ T-cell lines.

MEDLINE:96086826

**Constitutive NF-kappa B activation, enhanced granulopoiesis, and neonatal lethality in I kappa B alpha-deficient mice.**

Transcription factors belonging to the NF-kappa B family are controlled by inhibitory I kappa B proteins, mainly I kappa B alpha and I kappa B beta. Apparently normal at birth, I kappa B alpha-/- mice exhibit severe runting, skin defects, and extensive granulopoiesis postnatally, typically dying by 8 days. Hematopoietic tissues from these mice display elevated levels of both nuclear NF-kappa B and mRNAs of some, but not all, genes thought to be regulated by NF-kappa B. NF-kappa B elevation results in these phenotypic abnormalities because mice lacking both I kappa B alpha and the p50 subunit of NF-kappa B show a dramatically delayed onset of abnormalities. In contrast to hematopoietic cells, I kappa B alpha-/- embryonic fibroblasts show minimal constitutive NF-kappa B, as well as normal signal-dependent NF-kappa B activation that is concomitant with I kappa B beta degradation. Our results indicate that I kappa b beta, but not I kappa B alpha, is required for the signal-dependent activation of NF-kappa B in fibroblasts. However, I kappa B alpha is required for the postinduction repression of NF-kappa B in fibroblasts. These results define distinct roles for the two forms of I kappa B and demonstrate the necessity for stringent control of NF-kappa B.

MEDLINE:96085174

**Interleukin-7 can induce the activation of Jak 1, Jak 3 and STAT 5 proteins in murine T cells.**

The activation of Janus protein tyrosine kinases (Jak) and STAT (signal transducer and activator of transcription) proteins has recently been linked to the signal transduction mechanism of several cytokines. IL-7 was observed to induce a rapid and dose-dependent tyrosine phosphorylation of Jak 1 and Jak 3 and concomitantly, the tyrosine phosphorylation and DNA binding activity of multiple STAT proteins. The STAT proteins utilized by IL-7 were identical to those induced by IL-2 and could be identified as various STAT 5 isoforms. Moreover, the induction of both Jak 1 and 3, and STAT 5 activity strongly correlated with the growth-promoting effects of IL-7, suggesting that this signal transduction mechanism may play a key role in IL-7-induced proliferation.

MEDLINE:96009559

**N- and C-terminal sequences control degradation of MAD3/I kappa B alpha in response to inducers of NF-kappa B activity.**

The proteolytic degradation of the inhibitory protein MAD3/I kappa B alpha in response to extracellular stimulation is a prerequisite step in the activation of the transcription factor NF-kappa B. Analysis of the expression of human I kappa B alpha protein in stable transfectants of mouse 70Z/3 cells shows that, as for the endogenous murine protein, exogenous I kappa B alpha is degraded in response to inducers of NF-kappa B activity, such as phorbol myristate acetate or lipopolysaccharide. In addition, pretreatment of the cells with the proteasome inhibitor N-Ac-Leu-Leu-norleucinal inhibits this ligand-induced degradation and, in agreement with previous studies, stabilizes a hyperphosphorylated form of the human I kappa B alpha protein. By expressing mutant forms of the human protein in this cell line, we have been able to delineate the sequences responsible for both the ligand-induced phosphorylation and the degradation of I kappa B alpha. Our results show that deletion of the C terminus of the I kappa B alpha molecule up to amino acid 279 abolishes constitutive but not ligand-inducible phosphorylation and inhibits ligand-inducible degradation. Further analysis reveals that the inducible phosphorylation of I kappa B alpha maps to two serines in the N terminus of the protein (residues 32 and 36) and that the mutation of either residue is sufficient to abolish ligand-induced degradation, whereas both residues must be mutated to abolish inducible phosphorylation of the protein. (ABSTRACT TRUNCATED AT 250 WORDS)

MEDLINE:96074623

**Coexpression of NF-kappa B/Rel and Sp1 transcription factors in human immunodeficiency virus 1-induced, dendritic cell-T-cell syncytia.**

Productive infection of T cells with human immunodeficiency virus 1 (HIV-1) typically requires that the T cells be stimulated with antigens or mitogens. This requirement has been attributed to the activation of the transcription factor NF-kappa B, which synergizes with the constitutive transcription factor Sp1 to drive the HIV-1 promoter. Recently, we have found that vigorous replication of HIV-1 takes place in nonactivated memory T cells after syncytium formation with dendritic cells (DCs). These syncytia lack activated cells as determined by an absence of staining for Ki-67 cell cycle antigen. The expression and activity of NF-kappa B and Sp1 were, therefore, analyzed in isolated T cells and DCs from humans and mice. We have used immunolabeling, Western blot analysis, and electrophoretic mobility shift and supershift assays. T cells lack active NF-kappa B but express Sp1 as expected. DCs express high levels of all known NF-kappa B and Rel proteins, with activity residing primarily within RelB, p50, and p65. However, DCs lack Sp1, which may explain the failure of HIV-1 to replicate in purified DCs. Coexpression of NF-kappa B and Sp1 occurs in the heterologous DC-T-cell syncytia that are induced by HIV-1. Therefore, HIV-1-induced cell fusion brings together factors that upregulate virus transcription. Since DCs and memory T cells frequently traffic together in situ, these unusual heterologous syncytia could develop in infected individuals and lead to chronic HIV-1 replication without ostensible immune stimulation.

MEDLINE:96063646

**Cupric ion blocks NF kappa B activation through inhibiting the signal-induced phosphorylation of I kappa B alpha.**

A transcription factor NF kappa B, which regulates expression of various cellular genes involved in immune responses and viral genes including HIV, is sequestered in the cytoplasm as a complex with an inhibitory protein I kappa B. Various extracellular signals induce phosphorylation and rapid degradation of I kappa B alpha to release NF kappa B. Cu2+ was found to inhibit the activation of NF kappa B induced by TNF-alpha, TPA, or H2O2. Deoxycholate treatment of the cytoplasmic extract prepared from cells stimulated by TNF-alpha in the presence of Cu2+ resulted in the release of NF kappa B from I kappa B alpha, indicating that Cu2+ interferes with the dissociation of the NF kappa B-I kappa B complex. Neither phosphorylation nor degradation of I kappa B alpha was observed upon TNF-alpha stimulation in the presence of Cu2+. These results indicate that Cu2+ inhibits the release of NF kappa B by blockade of a signal leading to the phosphorylation of I kappa B alpha.

MEDLINE:96127450

**Activation of JAK3, but not JAK1, is critical for IL-2-induced proliferation and STAT5 recruitment by a COOH-terminal region of the IL-2 receptor beta-chain.**

A number of cytokines and growth factors use the JAK-STAT pathway to signal from the cell membrane to the nucleus. While homodimerizing cytokine receptors may transmit signal via a single form of JAK (i.e. growth hormone receptors), several multicomponent cytokine receptors have been shown to require simultaneous activation of pairs of different JAK kinases (i.e. interferon receptors). Recent evidence for a preferential coupling of JAK3 to interleukin-2 receptor-gamma (IL-2R gamma) and JAK1 to IL-2R beta supports the concept of heterotrans-activation of JAK1 and JAK3 caused by IL-2-induced heterodimerization of their receptor partners. The present study verified the ability of IL-2 to cause tyrosine phosphorylation and activation of JAK1 and JAK3, but demonstrated that IL-2 stimulated JAK3 to a significantly larger extent than JAK1 in human T lymphocytes and the YT cell line. This conclusion was based upon several independent criteria, including more vigorous tyrosine phosphorylation of JAK3, more marked enzymatic activation of JAK3 as well as higher abundance of JAK3 in activated IL-2 receptor complexes. Furthermore, when human IL-2R beta was stably expressed in murine BA/F3 cells, robust IL-2-induced proliferation and JAK3 activation occurred without detectable involvement of either JAK1, JAK2 or TYK2. We therefore propose that IL-2 receptor signal transduction does not depend on equimolar heterodimerization of JAK1 and JAK3 following IL-2-induced heterodimerization of IL-2R beta and IL-2R gamma. Nonetheless, a membrane-proximal region of human IL-2R beta (Asn240-Leu335) was critical for JAK3 activation, and the amount of JAK3 present in activated IL-2 receptor complexes increased with time, suggesting that stabilization of JAK3 binding to the receptor complex relies on both IL-2R beta and IL-2R gamma. Moreover, STAT5 was found to be the predominant STAT transcription factor used by IL-2 in human T cells, and specifically required a COOH-terminal region of IL-2R beta (Ser386-Val525), while STAT5 recruitment was not correlated to activation of IL-2R gamma or JAK3.

MEDLINE:95394931

**Ubiquitin-mediated processing of NF-kappa B transcriptional activator precursor p105. Reconstitution of a cell-free system and identification of the ubiquitin-carrier protein, E2, and a novel ubiquitin-protein ligase, E3, involved in conjugation.**

In most cases, the transcriptional factor NF-kappa B is a heterodimer consisting of two subunits, p50 and p65, which are encoded by two distinct genes of the Rel family. p50 is translated as a precursor of 105 kDa. The C-terminal domain of the precursor is rapidly degraded, forming the mature p50 subunit consisted of the N-terminal region of the molecule. The mechanism of generation of p50 is not known. It has been suggested that the ubiquitin-proteasome system is involved in the process; however, the specific enzymes involved and the mechanism of limited proteolysis, in which half of the molecule is spared, have been obscure. Palombella and colleagues (Palombella, V.J., Rando, O.J., Goldberg, A.L., and Maniatis, T.(1994) Cell 78, 773-785) have shown that ubiquitin is required for the processing in a cell-free system of a truncated, artificially constructed, 60-kDa precursor. They have also shown that proteasome inhibitors block the processing both in vitro and in vivo. In this study, we demonstrate reconstitution of a cell-free processing system and demonstrate directly that: (a) the ubiquitin-proteasome system is involved in processing of the intact p105 precursor, (b) conjugation of ubiquitin to the precursor is an essential intermediate step in the processing, (c) the recently discovered novel species of the ubiquitin-carrier protein, E2-F1, that is involved in the conjugation and degradation of p53, is also required for the limited processing of the p105 precursor, and (d) a novel, approximately 320-kDa species of ubiquitin-protein ligase, is involved in the process. This novel enzyme is distinct from E6-AP, the p53-conjugating ligase, and from E3 alpha, the "N-end rule" ligase.

MEDLINE:96125073

**A PEBP2 alpha/AML-1-related factor increases osteocalcin promoter activity through its binding to an osteoblast-specific cis-acting element.**

To identify osteoblast-specific cis-acting elements and trans-acting factors, we initiated an analysis of the promoter of a mouse osteocalcin gene, an osteoblast-specific gene. In this promoter, we identified two osteoblast-specific cis-acting elements (Ducy, P.and Karsenty, G.(1995) Mol.Cell.Biol.15, 1858-1869). The sequence of one of these elements, OSE2, is identical to the DNA-binding site of the PEBP2 alpha/AML-1 transcription factors, the mammalian homologues of the Drosophila Runt protein. Here we show, using nuclear extracts, recombinant protein, and a specific antiserum against AML-1 proteins in DNA-binding assays, that one member of this family, AML-1B, binds specifically to OSE2 and is immunologically related to OSF2, the factor present in osteoblast nuclear extracts that binds to OSE2. By DNA cotransfection experiments, we also demonstrate that AML-1B can increase the activity of a short osteocalcin promoter through its binding to OSE2. Lastly, the different mobilities of osteoblast nuclear extract-DNA complexes compared with T-cell nuclear extract-DNA complexes, along with the inability of OSF2 to be upregulated by retinoic acid, unlike the other PEBP2 alpha factors, suggest that OSF2 is a new member of this family of transcription factors. Thus, this study demonstrates that AML-1B can increase gene expression of an osteoblast-specific gene through its binding to an osteoblast-specific cis-acting element and presents evidence that OSF2 is a member of the PEBP2 alpha/AML-1 family of transcription factors.

MEDLINE:96070930

**Triggering of the human interleukin-6 gene by interferon-gamma and tumor necrosis factor-alpha in monocytic cells involves cooperation between interferon regulatory factor-1, NF kappa B, and Sp1 transcription factors.**

We investigated the molecular basis of the synergistic induction by interferon-gamma (IFN-gamma)/tumor necrosis factor-alpha (TNF-alpha) of human interleukin-6 (IL-6) gene in THP-1 monocytic cells, and compared it with the basis of this induction by lipopolysaccharide (LPS). Functional studies with IL-6 promoter demonstrated that three regions are the targets of the IFN-gamma and/or TNF-alpha action, whereas only one of these regions seemed to be implicated in LPS activation. The three regions concerned are: 1) a region between -73 and -36, which is the minimal element inducible by LPS or TNF-alpha; 2) an element located between -181 and -73, which appeared to regulate the response to IFN-gamma and TNF-alpha negatively; and 3) a distal element upstream of -224, which was inducible by IFN-gamma alone. LPS signaling was found to involve NF kappa B activation by the p50/p65 heterodimers. Synergistic induction of the IL-6 gene by IFN-gamma and TNF-alpha, in monocytic cells, involved cooperation between the IRF-1 and NF kappa B p65 homodimers with concomitant removal of the negative effect of the retinoblastoma control element present in the IL-6 promoter. This removal occurred by activation of the constitutive Sp1 factor, whose increased binding activity and phosphorylation were mediated by IFN-gamma.

MEDLINE:96055115

**Mutation of Jak3 in a patient with SCID: essential role of Jak3 in lymphoid development.**

Males with X-linked severe combined immunodeficiency (XSCID) have defects in the common cytokine receptor gamma chain (gamma c) gene that encodes a shared, essential component of the receptors of interleukin-2 (IL-2), IL-4, IL-7, IL-9, and IL-15. The Janus family tyrosine kinase Jak3 is the only signaling molecule known to be associated with gamma c, so it was hypothesized that defects in Jak3 might cause an XSCID-like phenotype. A girl with immunological features indistinguishable from those of XSCID was therefore selected for analysis. An Epstein-Barr virus (EBV)-transformed cell line derived from her lymphocytes had normal gamma c expression but lacked Jak3 protein and had greatly diminished Jak3 messenger RNA. Sequencing revealed a different mutation on each allele: a single nucleotide insertion resulting in a frame shift and premature termination in the Jak3 JH4 domain and a nonsense mutation in the Jak3 JH2 domain. The lack of Jak3 expression correlated with impaired B cell signaling, as demonstrated by the inability of IL-4 to activate Stat6 in the EBV-transformed cell line from the patient. These observations indicate that the functions of gamma c are dependent on Jak3 and that Jak3 is essential for lymphoid development and signaling.

MEDLINE:95394907

**Transcriptional regulation of the gene encoding the human C-type lectin leukocyte receptor AIM/CD69 and functional characterization of its tumor necrosis factor-alpha-responsive elements.**

The human activation antigen CD69 is a member of the C-type animal lectin superfamily that functions as a signal-transmitting receptor. Although the expression of CD69 can be induced in vitro on cells of most hematopoietic lineages with a wide variety of stimuli, in vivo it is mainly expressed by T-lymphocytes located in the inflammatory infiltrates of several human diseases. To elucidate the mechanisms that regulate the constitutive and inducible expression of CD69 by leukocytes, we isolated the promoter region of the CD69 gene and carried out its functional characterization. Sequence analysis of the 5'-flanking region of the CD69 gene revealed the presence of a potential TATA element 30 base pairs upstream of the major transcription initiation site and several putative binding sequences for inducible transcription factors (NF-kappa B, Egr-1, AP-1), which might mediate the inducible expression of this gene. Transient expression of CD69 promoter-based reporter gene constructs in K562 cells indicated that the proximal promoter region spanning positions -78 to +16 contained the cis-acting sequences necessary for basal and phorbol 12-myristate 13-acetate-inducible transcription of the CD69 gene. Removal of the upstream sequences located between positions -78 and -38 resulted in decreased promoter strength and abolished the response to phorbol 12-myristate 13-acetate. We also found that tumor necrosis factor-alpha (TNF-alpha) is capable of inducing the surface expression of the CD69 molecule as well as the promoter activity of fusion plasmids that contain 5'-flanking sequences of the CD69 gene, suggesting that this cytokine may regulate in vivo the expression of CD69. (ABSTRACT TRUNCATED AT 250 WORDS)

MEDLINE:95368765

**Characterization of 5' end of human thromboxane receptor gene. Organizational analysis and mapping of protein kinase C--responsive elements regulating expression in platelets.**

Platelet thromboxane receptors are acutely and reversibly upregulated after acute myocardial infarction. To determine if platelet thromboxane receptors are under transcriptional control, we isolated and characterized human genomic DNA clones containing the 5' flanking region of the thromboxane receptor gene. The exon-intron structure of the 5' portion of the thromboxane receptor gene was determined initially by comparing the nucleotide sequence of the 5' flanking genomic clone with that of a novel human uterine thromboxane receptor cDNA that extended the mRNA 141 bp further upstream than the previously identified human placental cDNA. A major transcription initiation site was located in three human tissues approximately 560 bp upstream from the translation initiation codon and 380 bp upstream from any previously identified transcription initiation site. The thromboxane receptor gene has neither a TATA nor a CAAT consensus site. Promoter function of the 5' flanking region of the thromboxane receptor gene was evaluated by transfection of thromboxane receptor gene promoter/chloramphenicol acetyltransferase (CAT) chimera plasmids into platelet-like K562 cells. Thromboxane receptor promoter activity, as assessed by CAT expression, was relatively weak but was significantly enhanced by phorbol ester treatment. Functional analysis of 5' deletion constructs in transfected K562 cells and gel mobility shift localized the major phorbol ester-responsive motifs in the thromboxane receptor gene promoter to a cluster of activator protein-2 (AP-2) binding consensus sites located approximately 1.8 kb 5' from the transcription initiation site. These studies are the first to determine the structure and organization of the 5' end of the thromboxane receptor gene and demonstrate that thromboxane receptor gene expression can be regulated by activation of protein kinase C via induction of an AP-2-like nuclear factor binding to upstream promoter elements. These findings strongly suggest that the mechanism for previously described upregulation of platelet thromboxane receptors after acute myocardial infarction is increased thromboxane receptor gene transcription in platelet-progenitor cells.

**Nuclear factor-IL6 activates the human IL-4 promoter in T cells.**

Positive regulatory element I (PRE-I) is a strong enhancer element essential for expression of the human IL-4 gene. To identify transcription factors binding to PRE-I, we screened a cDNA expression library from Jurkat T cells and isolated a cDNA encoding nuclear factor (NF)-IL6 (also known as C/EBP beta). NF-IL6 mRNA was found in human Jurkat T cells and in the mouse Th2 clone D10, but not in Th1 clone 29. rNF-IL6 expressed in bacteria was shown to specifically bind to PRE-I. PRE-I forms multiple DNA-protein complexes with nuclear extracts from Jurkat cells. Some of these complexes were demonstrated to contain NF-IL6 by using anti-C/EBP beta Abs. Overexpression of NF-IL6 enhanced expression of the chloramphenicol acetyl transferase reporter gene linked to the PRE-I-thymidine kinase or the human IL-4 promoter more than 10-fold in Jurkat cells. Promoter deletion studies revealed two additional NF-IL6 binding sites located at positions -44 to -36 (C/EBP proximal) and -87 to -79 (C/EBP medial), respectively. Our results demonstrate that NF-IL6 is involved in transcriptional activation of the human IL-4 promoter in T cells.

MEDLINE:96070929

**Identification of an I kappa B alpha-associated protein kinase in a human monocytic cell line and determination of its phosphorylation sites on I kappa B alpha.**

Nuclear factor kappa B (NF-kappa B) is stored in the cytoplasm as an inactive form through interaction with I kappa B. Stimulation of cells leads to a rapid phosphorylation of I kappa B alpha, which is presumed to be important for the subsequent degradation. We have recently reported the establishment of a lipopolysaccharide (LPS)-dependent cell-free activation system of NF-kappa B in association with the induction of I kappa B alpha phosphorylation. In this study, we have identified a kinase in cell extracts from the LPS-stimulated human monocytic cell line, THP-1, that specifically binds and phosphorylates I kappa B alpha. LPS stimulation transiently enhanced the I kappa B alpha-bound kinase activity in THP-1 cells. Mutational analyses of I kappa B alpha and competition experiments with the synthetic peptides identified major phosphorylation sites by the bound kinase as Ser and Thr residues in the C-terminal acidic domain of I kappa B alpha. Moreover, we show that the peptide, corresponding to the C-terminal acidic domain of I kappa B alpha, blocked the LPS-induced NF-kappa B activation as well as inducible phosphorylation of endogenous I kappa B alpha in a cell-free system using THP-1 cells. These results suggested that the bound kinase is involved in the signaling pathway of LPS by inducing the phosphorylation of the C-terminal region of I kappa B alpha and subsequent dissociation of the NF-kappa B.I kappa B alpha complex.

MEDLINE:96068922

**Bik, a novel death-inducing protein shares a distinct sequence motif with Bcl-2 family proteins and interacts with viral and cellular survival-promoting proteins.**

The survival-promoting activity of the Bcl-2 family of proteins appears to be modulated by interactions between various cellular proteins. We have identified a novel cellular protein, Bik, that interacts with the cellular survival-promoting proteins, Bcl-2 and Bcl-xL, as well as the viral survival-promoting proteins, Epstein Barr virus-BHRF1 and adenovirus E1B-19 kDa. In transient transfection assays, Bik promotes cell death in a manner similar to the death-promoting members of the Bcl-2 family, Bax and Bak. This death-promoting activity of Bik can be suppressed by coexpression of Bcl-2, Bcl-XL, EBV-BHRF1 and E1B-19 kDa proteins suggesting that Bik may be a common target for both cellular and viral anti-apoptotic proteins. While Bik does not show overt homology to the BH1 and BH2 conserved domains characteristic of the Bcl-2 family, it does share a 9 amino acid domain (BH3) with Bax and Bak which may be a critical determinant for the death-promoting activity of these proteins.

MEDLINE:96128660

**Cross-linking of Fc gamma receptors activates HIV-1 long terminal repeat-driven transcription in human monocytes.**

Elevation of the levels of circulating immune complexes frequently accompanies HIV-1 infection and is a prognostic indicator of clinical progression from asymptomatic infection to AIDS. Here we report that cross-linking of Fc gamma RI or Fc gamma RII by adherent human IgG or by specific anti-Fc gamma R mAb activates HIV-1 gene expression in the human monocytic cell line BF24 and increased HIV RNA expression in monocytes from HIV infected patients as assayed by reverse transcription-PCR. In THP-1 cells, Fc gamma R cross-linking induced NF-kappa B, which is known to bind to the regulatory region of the long terminal repeat (LTR) of HIV-1 and to activate HIV-1 transcription. Anti-TNF-alpha antibody but not anti-IL-1 beta antibody strongly inhibited both the induction of HIV-1-LTR-driven transcription and the induction of NF-kappa B by Fc gamma R cross-linking. These results indicate that Fc gamma R can mediate a TNF-alpha-dependent induction of HIV-1 gene transcription and suggest that immune complexes may contribute to the pathophysiology of HIV-1 infection by augmenting viral replication in monocytes.

MEDLINE:95363941

**C/EBP proteins activate transcription from the human immunodeficiency virus type 1 long terminal repeat in macrophages/monocytes.**

Three binding sites for C/EBP proteins are found in the human immunodeficiency virus type 1 (HIV-1) long terminal repeat (LTR) (V.M. Tesmer, A.Rajadhyaksha, J.Babin, and M.Bina, Proc.Natl.Acad.Sci. USA 90:7298-7302, 1993). We have determined the functional role of C/EBP proteins and C/EBP sites in regulating transcription from the HIV- 1 LTR in monocytes/macrophages. Inhibition of endogenous C/EBP proteins, using either an excess of C/EBP binding sites or a trans- dominant negative inhibitor, demonstrated that C/EBP proteins are required for basal and activated levels of HIV-1 LTR transcription in the promonocytic cell line U937. Northern (RNA) blots and binding assays showed that NF-IL6 is the only known C/EBP family member which is increased when U937 cells are activated. Mutational analyses of the HIV-1 LTR showed that one C/EBP site is required for normal LTR transcription both before and after cellular activation and that the two 3' C/EBP sites are functionally equivalent. However, transcription from crippled HIV-1 LTRs lacking C/EBP sites can still be induced following activation of U937 cells. Several models are suggested for how elevated NF-IL6 may participate in an autostimulatory loop involving HIV infection, macrophage activation, cytokine expression, and HIV replication.

MEDLINE:96079922

**Prolactin and interleukin-2 receptors in T lymphocytes signal through a MGF-STAT5-like transcription factor.**

The cell surface receptors for PRL and interleukin-2 (IL-2) are structurally distinct, but share regulatory tasks in T lymphocytes. They can stimulate proliferation and activate transcription of over-lapping sets of genes of T cells. PRL and IL-2 receptor activation are both linked to the Jak/Stat (signal transducer and activator of transcription) pathway. We investigated the ability of PRL and IL-2 to activate Stat proteins in different T cell lines. The DNA binding specificities, the reactivities toward Stat-specific antisera, and the mol wt of IL-2 -and PRL-induced DNA-binding proteins in Nb2 and C196 T cell lines were investigated. A comparison with the Stat proteins induced by interferon-gamma, PRL, and IL-6 in T47D mammary tumor cells was made. We found that these parameters were indistinguishable for one of the PRL- and IL-2-induced factors. A transcription factor closely related to mammary gland factor-Stat5 is rapidly activated upon interaction of IL-2 and PRL with their respective receptors. Activation of a second protein related to Stat1 was also observed. Our results emphasize the role of PRL as a regulator of the immune response and indicate that the Stat factors mammary gland factor-Stat5 and Stat1 play a role in the regulation of gene expression during T cell development.

MEDLINE:96074843

**ETS1 transactivates the human GM-CSF promoter in Jurkat T cells stimulated with PMA and ionomycin.**

Activation of T helper cells results in coordinate expression of a number of cytokines involved in differentiation, proliferation and activation of the haematopoietic system. Granulocyte-macrophage colony-stimulating factor (GM-CSF) is one such cytokine whose increased expression results partly from increases in transcription. Cis-acting elements with NF kappa B, AP-1 and ETS-like motifs have been identified in the promoter region of the GM-CSF gene, which are important for transcriptional activity following PMA and ionomycin stimulation. A number of the ETS family of transcription factors are expressed in T cells, including ETS1 and ELF1. Here we describe the ability of these factors to interact with a site (GM5), located within the CLE0 element, -47 to -40 upstream of the GM-CSF transcription initiation site. Exogenous ETS1, but not ELF1, can transactivate GM-CSF, through the GM5 site, in a PMA/ionomycin dependent manner. Other unidentified ETS-like factors present in Jurkat cells are also capable of binding GM5. Mutation of the core ETS binding site from -GGAA- to -GGAT- prevents the binding of ETS-like factors with the exception of ETS1. The GM-CSF promoter, modified in this way to be ETS1 specific, is fully responsive to PMA/ionomycin induction, in addition to ETS1 transactivation in the presence of PMA and ionomycin. Together these data suggest that ETS1 may be involved in mediating the increased GM-CSF production associated with T cell activation.

MEDLINE:96379940

**Quantitation of beta 1 triiodothyronine receptor mRNA in human tissues by competitive reverse transcription polymerase chain reaction.**

Thyroid hormones act by binding to nuclear receptor proteins, the thyroid hormone receptors (TR) alpha and beta. Data from cell culture and animal studies indicate that TR expression may be regulated to modulate target organ responsiveness to thyroid hormone. To investigate whether such adaptive changes in TR expression occur in humans, we determined the mRNA levels of the hTR beta 1 in various thyroid states. Patients with overt hypo -or hyperthyroidism were enrolled in the study. Total RNA was isolated from peripheral blood mononuclear cells and hTR beta 1 mRNA levels determined by quantitative competitive reverse transcription PCR. For comparison, hTR beta 1 mRNA levels were determined in lymphocytes and normal thyroid tissue of euthyroid patients. Human TR beta 1 mRNA levels in lymphocytes were 1.8 +/- 0.4, 1.9 +/- 0.5, 1.1 +/- 0.4 10(-18) mol/microgram RNA in hypo-, eu -and hyperthyroid patients, respectively, corresponding to an estimated 0.5 -2 molecules per cell. Although the mean hTR beta 1 mRNA levels were 40% lower in hyperthyroid than in euthyroid subjects, this difference did not reach statistical significance. Similar levels of hTR beta 1 mRNA levels were detected in thyroid gland from euthyroid patients. In summary, we developed an assay for the quantitative determination of hTR beta 1 mRNA levels in small human tissue samples, containing as little as 50 ng of total RNA. Absolute hTR beta 1 mRNA levels are very low with an estimated one molecule of mRNA being present in a mononuclear blood cell or thyrocyte. No up-regulation of hTR beta 1 was seen in hypothyroid relative to euthyroid patients. However, there is a non-significant trend towards a down-regulation of hTR beta 1 mRNA levels in hyperthyroid patients.

MEDLINE:95399771

**Regulation of granulocyte-macrophage colony-stimulating factor and E-selectin expression in endothelial cells by cyclosporin A and the T-cell transcription factor NFAT.**

Nuclear factor of activated T cells (NFAT) was originally described as a T-cell-specific transcription factor athat supported the activation of cytokine gene expression and mediated the immunoregulatory effects of cyclosporin A (CsA). As we observed that activated endothelial cells also expressed NFAT, we tested the antiinflammatory properties of CsA in endothelial cells. Significantly, CsA completely suppressed the induction of NFAT in endothelial cells and inhibited the activity of granulocyte-macrophage colony-stimulating factor (GM-CSF) gene regulatory elements that use NFAT by 60%. CsA similarly mediated a reduction of up to 65% in GM-CSF mRNA and protein expression in activated endothelial cells. CsA also suppressed E-selectin, but not vascular cell adhesion molecule-1 (VCAM-1) expression in endothelial cells, even though the E-selectin promoter is activated by NF-kappa B rather than NFAT. Hence, induction of cell surface expression of this leukocyte adhesion molecule by tumor necrosis factor (TNF)-alpha was reduced by 40% in the presence of CsA, and this was reflected by a 29% decrease in neutrophil adhesion. The effects of CsA on endothelial cells were also detected at the chromatin structure level, as DNasel hypersensitive sites within both the GM-CSF enhancer and the E-selectin promoter were suppressed by CsA. This represents the first report of NFAT in endothelial cells and suggests mechanisms by which CsA could function as an antiinflammatory agent.

MEDLINE:96049646

**Costimulation requirement for AP-1 and NF-kappa B transcription factor activation in T cells.**

The transcriptional activity of the IL-2 promoter requires T-cell costimulation delivered by the TCR and the auxiliary receptor CD28. Several transcription factors participate in IL-2 promoter activation, among which are AP-1-like factors and NF-kappa B. Protein phosphorylation has an important role in the regulation of these two factors: (1) it induces the transactivating capacity of the AP-1 protein c-Jun; and (2) it is involved in the release of the cytoplasmic inhibitor, I kappa B, from NF-kappa B, allowing translocation of the latter into the nucleus. We have recently shown that both phosphorylation processes require T-cell costimulation. Furthermore, in activated T cells, the kinetics of the two phosphorylation events are essentially similar. According to our results, however, the kinases responsible for the two processes are distinct entities. Whereas TPCK inhibits phosphorylation of I kappa B and, consequently, activation of NF-kappa B, it markedly enhances the activity of JNK, the MAP kinase-related kinase that phosphorylates the transactivation domain of c-Jun. We, therefore, propose the activation scheme presented in FIGURE 3 for T-cell costimulation. Costimulation results in the activation of a signaling pathway that leads to the simultaneous induction of the two transcription factors, AP-1 and NF-kappa B. Integration of the signals generated by TCR and CD28 engagement occurs along this pathway, which then bifurcates to induce I kappa B phosphorylation and NF-kappa B activation on the one hand, and JNK activation and c-Jun phosphorylation on the other. We are currently engaged in defining where the two signals integrate along the AP-1/NF-kappa B pathway.

MEDLINE:96035700

**Regulation of human immunodeficiency virus type 1 and cytokine gene expression in myeloid cells by NF-kappa B/Rel transcription factors.**

CD4+ macrophages in tissues such as lung, skin, and lymph nodes, promyelocytic cells in bone marrow, and peripheral blood monocytes serve as important targets and reservoirs for human immunodeficiency virus type 1 (HIV-1) replication. HIV-1-infected myeloid cells are often diminished in their ability to participate in chemotaxis, phagocytosis, and intracellular killing. HIV-1 infection of myeloid cells can lead to the expression of surface receptors associated with cellular activation and/or differentiation that increase the responsiveness of these cells to cytokines secreted by neighboring cells as well as to bacteria or other pathogens. Enhancement of HIV-1 replication is related in part to increased DNA-binding activity of cellular transcription factors such as NF-kappa B. NF-kappa B binds to the HIV-1 enhancer region of the long terminal repeat and contributes to the inducibility of HIV-1 gene expression in response to multiple activating agents. Phosphorylation and degradation of the cytoplasmic inhibitor I kappa B alpha are crucial regulatory events in the activation of NF-kappa B DNA-binding activity. Both N- and C-terminal residues of I kappa B alpha are required for inducer-mediated degradation. Chronic HIV-1 infection of myeloid cells leads to constitutive NF-kappa B DNA-binding activity and provides an intranuclear environment capable of perpetuating HIV-1 replication. Increased intracellular stores of latent NF-kappa B may also result in rapid inducibility of NF-kappa B-dependent cytokine gene expression. In response to secondary pathogenic infections or antigenic challenge, cytokine gene expression is rapidly induced, enhanced, and sustained over prolonged periods in HIV-1-infected myeloid cells compared with uninfected cells. Elevated levels of several inflammatory cytokines have been detected in the sera of HIV-1-infected individuals. Secretion of myeloid cell-derived cytokines may both increase virus production and contribute to AIDS-associated disorders.

MEDLINE:96102041

**Functional characterization of the murine homolog of the B cell-specific coactivator BOB.1/OBF.1.**

B cell-specific transcriptional promoter activity mediated by the octamer motif requires the Oct1 or Oct2 protein and additional B cell-restricted cofactors. One such cofactor, BOB.1/OBF.1, was recently isolated from human B cells. Here, we describe the isolation and detailed characterization of the murine homolog. Full-length cDNAs and genomic clones were isolated, and the gene structure was determined. Comparison of the deduced amino acids shows 88% sequence identity between mouse and human BOB.1/OBF.1. The NH2-terminal 126 amino acids of BOB.1/OBF.1 are both essential and sufficient for interaction with the POU domains of either Oct1 or Oct2. This protein-protein interaction does not require the simultaneous binding of Oct proteins to DNA, and high resolution footprinting of the Oct-DNA interaction reveals that binding of BOB.1/OBF.1 to Oct1 or Oct2 does not alter the interaction with DNA. BOB.1/OBF.1 can efficiently activate octamer-dependent promoters in fibroblasts; however, it fails to stimulate octamer-dependent enhancer activity. Fusion of subdomains of BOB.1/OBF.1 with the GAL4 DNA binding domain reveals that both NH2- and COOH-terminal domains of BOB.1/OBF.1 contribute to full transactivation function, the COOH-terminal domain is more efficient in this transactivation assay. Consistent with the failure of full-length BOB.1/OBF.1 to stimulate octamer-dependent enhancer elements in non B cells, the GAL4 fusions likewise only stimulate from a promoter-proximal position.

MEDLINE:96062038

**CD30 ligation induces nuclear factor-kappa B activation in human T cell lines.**

CD30 is a recently described member of the tumor necrosis factor/nerve growth factor receptor superfamily. In this report, we show that following incubation of L540 cells (Hodgkin's disease-derived, T cell-like, CD30+ cells) with the agonistic anti-CD30 monoclonal antibodies (mAb) M44 and M67, two nuclear factor (NF)-kappa B DNA binding activities were induced in nuclear extracts, as determined in gel retardation assays. The effect of the mAb towards NF-kappa B activation was rapid, as it occurred within 20 min, and was sustained for up to 6 h. By comparison, an isotype-matched antibody had no effect on NF-kappa B activation. Moreover, in human T helper (Th) clones functionally characterized as being of the type 0, type 1 and type 2 (28%, < 1% und 93% CD30+, respectively), the extent of CD30-mediated NF-kappa B activation correlated with the proportion of CD30+ cells. In all cell lines investigated, the NF-kappa B complexes induced following CD30 engagement were shown to contain p50 NF-kappa B1, p65 RelA, and possibly other transcription factors. Collectively, our results demonstrate that nuclear translocation and activation of NF-kappa B rank among the short-term cellular responses elicited following CD30 ligation.

MEDLINE:90110496

**Anti-Ro(SSA) autoantibodies are associated with T cell receptor beta genes in systemic lupus erythematosus patients.**

Several of the heterogeneous clinical manifestations of systemic lupus erythematosus have been associated with specific autoantibodies. Associations between HLA class II antigens and autoantibodies to the ribonucleoproteins Ro(SSA) and La(SSB) have been reported in these patients. Because HLA class II molecules present antigen to T cell receptors (TCRs), we have searched for a TCR gene associated with the production of anti-Ro(SSA) antibodies. A pair of restriction fragment length polymorphisms (RFLPs), one of which hybridizes to the TCR constant region C beta 1 and the other to the C beta 2 gene, has been identified, suggesting these may be genotypic markers for an extended region of the TCR beta locus. This RFLP pair occurs in 76% of patients with Ro(SSA) precipitins, 84% of anti-Ro(SSA)-positive patients lacking La(SSB) precipitins, but only 41% of the patients lacking both precipitins (P = 0.0004). This disproportionate occurrence in a subset of lupus patients indicates that these RFLPs are not disease susceptibility markers, but rather are important markers for TCR genes whose products are involved in the production of anti-Ro(SSA) antibodies. The majority of patients who have these RFLPs and HLA class II antigens previously associated with the anti-Ro(SSA) response make this antibody, suggesting that interactions between products of these loci occur in response to Ro(SSA).

MEDLINE:96102166

**Inhibition of NF-AT-dependent transcription by NF-kappa B: implications for differential gene expression in T helper cell subsets.**

Activation of individual CD4+ T cells results in differential lymphokine expression: interleukin 2 (IL-2) is preferentially produced by T helper type 1 (TH1) cells, which are involved in cell-mediated immune responses, whereas IL-4 is synthesized by TH2 cells, which are essential for humoral immunity. The Ca(2+)-dependent factor NF-ATp plays a key role in the inducible transcription of both these lymphokine genes. However, while IL2 expression requires the contribution of Ca(2+)- and protein kinase C-dependent signals, we report that activation of human IL4 transcription through the Ca(2+)-dependent pathway is diminished by protein kinase C stimulation in Jurkat T cells. This phenomenon is due to mutually exclusive binding of NF-ATp and NF-kappa B to the P sequence, an element located 69 bp upstream of the IL4 transcription initiation site. Human IL4 promoter-mediated transcription is downregulated in Jurkat cells stimulated with the NF-kappa B-activating cytokine tumor necrosis factor alpha and suppressed in RelA-overexpressing cells. In contrast, protein kinase C stimulation or RelA overexpression does not affect the activity of a human IL4 promoter containing a mouse P sequence, which is a higher-affinity site for NF-ATp and a lower-affinity site for RelA. Thus, competition between two general transcriptional activators, RelA and NF-ATp, mediates the inhibitory effect of protein kinase C stimulation on IL4 expression and may contribute to differential gene expression in TH cells.

MEDLINE:96096457

**Regulation of the balance of cytokine production and the signal transducer and activator of transcription (STAT) transcription factor activity by cytokines and inflammatory synovial fluids.**

The balance between type 1 and 2 T helper cell cytokine production plays an important role in several animal models of autoimmunity, and skewed patterns of cytokine expression have been described in human inflammatory diseases. Many cytokines activate signal transducer and activation of transcription (STAT) transcription factors, which, in turn, activate transcription of inflammatory effector genes. We used mononuclear cell priming cultures and inflammatory synovial fluids (SFs) derived from arthritis patients to examine the regulation of cytokine production and STAT activity by an inflammatory synovial microenvironment. Exposure to SFs during priming resulted in an 81% inhibition of interferon (IFN)-gamma, but not interleukin (IL) 4, production by effector cells generated in priming cultures. SF suppression was mediated by IL-4 and IL-10 and inhibition of IL-12 expression, and it was reversed in a dominant fashion by exogenous IL-12. SFs blocked the sustained activity of transcription factor Stat1, but not Stat3, during the priming period, and Stat1 activity was differentially regulated by cytokines in parallel with their positive or negative regulation of IFN-gamma production. Active Stat3, but not Stat1, was detected in cells from inflamed joints. These results suggest a role for altered balance of Stat1 and Stat3 transcriptional activity in the regulation of T cell differentiation and in the pathogenesis of inflammatory synovitis.

MEDLINE:96062305

**Triggering of complement receptors CR1 (CD35) and CR3 (CD11b/CD18) induces nuclear translocation of NF-kappa B (p50/p65) in human monocytes and enhances viral replication in HIV-infected monocytic cells.**

Monocyte/macrophages may harbor HIV in a nonproductive fashion for prolonged periods of time. Viral gene expression may be reactivated by stimulation of the cells with LPS or cytokines such as TNF-alpha in vitro. The effect of LPS and TNF-alpha is mediated by their ability to induce nuclear translocation of the DNA-binding heterodimer NF-kappa B (p50/p65), which binds to a specific sequence in the HIV-long terminal repeat. The present study demonstrates that triggering of complement receptors CR1 (CD35) and CR3 (CD11b/CD18) enhances viral replication in HIV-infected human monocytic cells. Monocytic cell lines and normal peripheral blood monocytes were infected with HIV-1 in vitro and cultured in the presence or absence of F(ab')2 fragments of monoclonal anti-CR1 or anti-CR3 Abs or with C3 fragments. Stimulation of CR1 or CR3 induces a two- to fourfold increase in the amount of cell-associated and released p24 Ag in cell cultures that was equivalent to that observed in control cultures triggered with LPS. We further observed that stimulation of CR1 or CR3 induces the nuclear translocation of NF-kappa B p50/p65 in infected cells. Translocation of NF-kappa B p50/p65 was also observed following stimulation of CR1 or CR3 of uninfected peripheral blood monocytes from HIV-seronegative donors. The amount of protein translocated was similar to that observed when cells were stimulated with rhTNF-alpha. TNF-alpha did not mediate the translocation of NF-kappa B p50/p65 induced by triggering of complement receptors. Taken together, these observations suggest that HIV gene expression may be activated in infected monocytes through interaction of the cells with complement-opsonized particles and that enhanced viral replication is associated with C3 receptor-mediated nuclear translocation of the NF-kappa B complex.

MEDLINE:96025779

**Mutually exclusive interaction of a novel matrix attachment region binding protein and the NF-muNR enhancer repressor. Implications for regulation of immunoglobulin heavy chain expression.**

The immunoglobulin heavy chain (IgH) intronic enhancer stimulates transcription from functional promoters in B lymphocytes but not other cell types. The observation that binding sites for the nuclear factor-mu negative regulator (NF-muNR) enhancer repressor overlap nuclear matrix attachment regions (MARs) in this enhancer has lead to the hypothesis that the cell type specificity of the enhancer might be controlled by regulating nuclear matrix attachment (Scheuermann, R. H., and Chen, U. (1989) Genes & Dev. 3, 1255-1266). To understand the role of MARs in IgH enhancer regulation, we have identified a novel MAR-binding protein, MAR-BP1, from soluble nuclear matrix preparations based on its ability to bind to the MARs associated with the IgH enhancer. Purified MAR-BP1 migrates as a 33-kDa protein, and it can be found in nuclear matrix preparations from a number of different types of lymphoid cell lines. Although specific binding sites have been difficult to localize by chemical or enzymatic footprinting procedures, NF-muNR binding sites are critical for efficient MAR-BP1 binding. Indeed, binding of the IgH enhancer to either intact nuclear matrix preparations or to MAR-BP1 is mutually exclusive to NF-muNR binding. These results are consistent with a model for cell-type specific regulation in which binding of the NF-muNR repressor to the IgH enhancer prevents nuclear matrix attachment in inappropriate cells by interfering with MAR-BP1/enhancer interaction.

MEDLINE:96009612

**PU.1 (Spi-1) and C/EBP alpha regulate expression of the granulocyte-macrophage colony-stimulating factor receptor alpha gene.**

Growth factor receptors play an important role in hematopoiesis. In order to further understand the mechanisms directing the expression of these key regulators of hematopoiesis, we initiated a study investigating the transcription factors activating the expression of the granulocyte-macrophage colony-stimulating factor (GM-CSF) receptor alpha gene. Here, we demonstrate that the human GM-CSF receptor alpha promoter directs reporter gene activity in a tissue-specific fashion in myelomonocytic cells, which correlates with its expression pattern as analyzed by reverse transcription PCR. The GM-CSF receptor alpha promoter contains an important functional site between positions -53 and -41 as identified by deletion analysis of reporter constructs. We show that the myeloid and B cell transcription factor PU.1 binds specifically to this site. Furthermore, we demonstrate that a CCAAT site located upstream of the PU.1 site between positions -70 and -54 is involved in positive-negative regulation of the GM-CSF receptor alpha promoter activity. C/EBP alpha is the major CCAAT/enhancer-binding protein (C/EBP) form binding to this site in nuclear extracts of U937 cells. Point mutations of either the PU.1 site or the C/EBP site that abolish the binding of the respective factors result in a significant decrease of GM-CSF receptor alpha promoter activity in myelomonocytic cells only. Furthermore, we demonstrate that in myeloid and B cell extracts, PU.1 forms a novel, specific, more slowly migrating complex (PU-SF) when binding the GM-CSF receptor alpha promoter PU.1 site. This is the first demonstration of a specific interaction with PU.1 on a myeloid PU.1 binding site. The novel complex is distinct from that described previously as binding to B cell enhancer sites and can be formed by addition of PU.1 to extracts from certain nonmyeloid cell types which do not express PU.1, including T cells and epithelial cells, but not from erythroid cells. Furthermore, we demonstrate that the PU-SF complex binds to PU.1 sites found on a number of myeloid promoters, and its formation requires an intact PU.1 site adjacent to a single-stranded region. Expression of PU.1 in nonmyeloid cells can activate the GM-CSF receptor alpha promoter. Deletion of the amino-terminal region of PU.1 results in a failure to form the PU-SF complex and in a concomitant loss of transactivation, suggesting that formation of the PU-SF complex is of functional importance for the activity of the GM-CSF receptor alpha promoter. Finally, we demonstrate that C/EBP alpha can also active the GM-CSF receptor alpha promoter in nonmyeloid cells. (ABSTRACT TRUNCATED AT 400 WORDS)

MEDLINE:96024587

**HMG-I binds to GATA motifs: implications for an HPFH syndrome.**

We have examined binding of the nuclear protein HMG-I to the human gamma-globin promoter. We find that HMG-I binds preferentially to the more 3' of a pair of GATA motifs in the gamma-globin promoter; this paired motif is bound by the erythroid factor GATA-1. A naturally occurring mutation (-175 T-C) in the area bound by HMG-I results in overexpression of gamma-globin in adult red blood cells (HPFH) and up-regulation of the gamma-globin promoter in in vitro expression assays; HMG-I does not bind to this mutant sequence. A survey of GATA motifs from other globin cis-elements demonstrates HMG-I binding to most of them. These findings implicate HMG-I in the HPFH phenotype; we speculate that it may participate in the formation of multiprotein complexes that regulate globin gene expression.

MEDLINE:96026328

**Immunosuppression by glucocorticoids: inhibition of NF-kappa B activity through induction of I kappa B synthesis [see comments]**

Glucocorticoids are among the most potent anti-inflammatory and immunosuppressive agents. They inhibit synthesis of almost all known cytokines and of several cell surface molecules required for immune function, but the mechanism underlying this activity has been unclear. Here it is shown that glucocorticoids are potent inhibitors of nuclear factor kappa B (NF-kappa B) activation in mice and cultured cells. This inhibition is mediated by induction of the I kappa B alpha inhibitory protein, which traps activated NF-kappa B in inactive cytoplasmic complexes. Because NF-kappa B activates many immunoregulatory genes in response to pro-inflammatory stimuli, the inhibition of its activity can be a major component of the anti-inflammatory activity of glucocorticoids.

MEDLINE:96009608

**Transcriptional repression of the interleukin-2 gene by vitamin D3: direct inhibition of NFATp/AP-1 complex formation by a nuclear hormone receptor.**

T-lymphocyte proliferation is suppressed by 1,25-dihydroxyvitamin D3 [1,25(OH)2D3], the active metabolite of vitamin D3, and is associated with a decrease in interleukin 2 (IL-2), gamma interferon, and granulocyte-macrophage colony-stimulating factor mRNA levels. We report here that 1,25(OH)2D3-mediated repression in Jurkat cells is cycloheximide resistant, suggesting that it is a direct transcriptional repressive effect on IL-2 expression by the vitamin D3 receptor (VDR). We therefore examined vitamin D3-mediated repression of activated IL-2 expression by cotransfecting Jurkat cells with IL-2 promoter/reporter constructs and a VDR overexpression vector and by DNA binding. We delineated an element conferring both DNA binding by the receptor in vitro and 1,25(OH)2D3-mediated repression in vivo to a short 40-bp region encompassing an important positive regulatory element, NF-AT-1, which is bound by a T-cell-specific transcription factor, NFATp, as well as by AP-1. VDR DNA-binding mutants were unable to either bind to this element in vitro or repress in vivo; the VDR DNA-binding domain alone, however, bound the element but also could not repress IL-2 expression. These results indicate that DNA binding by VDR is necessary but not sufficient to mediate IL-2 repression. By combining partially purified proteins in vitro, we observed the loss of the bound NFATp/AP-1-DNA complex upon inclusion of VDR or VDR-retinoid X receptor. Order of addition and off-rate experiments indicate that the VDR-retinoid X receptor heterodimer blocks NFATp/AP-1 complex formation and then stably associates with the NF-AT-1 element. This direct inhibition by a nuclear hormone receptor of transcriptional activators of the IL-2 gene may provide a mechanistic explanation of how vitamin derivatives can act as potent immunosuppressive agents.

MEDLINE:95386516

**Phosphorylation of the transcription factor NFATp inhibits its DNA binding activity in cyclosporin A-treated human B and T cells.**

Cyclosporin A (CsA) exerts its immunosuppressive effect by inhibiting the activity of nuclear factor of activated T cells (NFAT), thus preventing transcriptional induction of several cytokine genes. This effect is thought to be largely mediated through inactivation of the phosphatase calcineurin, which in turn inhibits translocation of an NFAT component to the nucleus. Here we report that CsA treatment of Raji B and Jurkat T cell lines yields a phosphorylated form of NFATp that is inhibited in DNA-binding and in its ability to form an NFAT complex with Fos and Jun. Immunoblot analyses and metabolic labeling with [32P]orthophosphate show that CsA alters NFATp migration on SDS-polyacrylamide gel electrophoresis by increasing its phosphorylation level without affecting subcellular distribution. Dephosphorylation by in vitro treatment with calcineurin or alkaline phosphatase restores NFATp DNA binding activity and its ability to reconstitute an NFAT complex with Fos and Jun proteins. These data point to a new mechanism for CsA-sensitive regulation of NFATp in which dephosphorylation is critical for DNA binding.

MEDLINE:96226053

**Expression of Id2 and Id3 mRNA in human lymphocytes.**

Helix-loop-helix (HLH) transcription factors are involved in cellular growth and differentiation. The Id (inhibitor of DNA binding and differentiation) HLH proteins, in a dominantly negative fashion, regulate transcriptional activities of basic HLH proteins. We examined by northern hybridization the expression of Id2 and Id3 mRNA in human leukemia/lymphoma lines and patient samples, as well as resting and activated normal human lymphocytes from peripheral blood (PBL). The Id2 mRNA was abundantly expressed in 5/12 T-cell and 3/4 B-cell lines, and Id3 mRNA was detected in 4/12 T-cell and 3/4 B-cell lines. Interestingly, Id2, but not Id3, mRNA was strongly expressed in 4/5 T-cell lines infected with human T-cell leukemia virus type I (HTLV-I) (ATL-1k, MT-2, S-LB1) and type II (Mo). Another unexpected finding was that T-cell leukemias and T-cell lines often expressed either Id2 or Id3 mRNA. In addition, resting PBL constitutively expressed prominent levels of Id2 mRNA, but not Id3 mRNA. Upon PHA-stimulation, Id2 expression decreased and Id3 levels increased with biphasic kinetics. Taken together, our studies revealed three unexpected findings which require further analysis: (1) expression of Id2 mRNA is often associated with lymphocytic transformation by HTLV-I or -II; (2) T-cells usually express either Id2 or Id3 mRNA, but B-cells often express both simultaneously; (3) non-dividing, normal PBL express high levels of Id2 and no Id3 mRNA; and with the onset of cellular proliferation, levels of Id2 mRNA decrease while levels of Id3 mRNA increase, suggesting that regulation of expression of these closely related genes is disparate.

MEDLINE:96082168

**Salicylates inhibit lipopolysaccharide-induced transcriptional activation of the tissue factor gene in human monocytic cells.**

Binding of plasma Factor VII/VIIa to the tissue factor (TF) receptor initiates the coagulation protease cascades. TF expression by circulating monocytes is associated with thrombotic and inflammatory complications in a variety of diseases. Transcriptional activation of the human TF gene in monocytic cells exposed to bacterial lipopolysaccharide (LPS) is mediated by binding of c-Rel/p65 heterodimers to a kappa B site in the TF promoter. Here, we report that a family of anti-inflammatory agents, known as the salicylates, inhibited LPS induction of TF activity and TF gene transcription in human monocytes and monocytic THP-1 cells at clinically relevant doses. Furthermore, sodium salicylate blocked the LPS-induced proteolytic degradation of I kappa B alpha, which prevented the nuclear translocation of c-Rel/p65 heterodimers. In contrast, two other nonsteroidal anti-inflammatory drugs, ibuprofen and indomethacin, did not inhibit LPS induction of the TF gene. These results indicated that salicylates inhibited LPS induction of TF gene transcription in monocytic cells by preventing nuclear translocation of c-Rel/p65 heterodimers. The clinical benefits of salicylates in the treatment of several diseases, including atherosclerosis and rheumatoid arthritis, may be related to their ability to reduce monocyte gene expression.

MEDLINE:95403454

**A regulatory element in the human interleukin 2 gene promoter is a binding site for the zinc finger proteins Sp1 and EGR-1.**

Activation of the interleukin 2 (IL-2) gene after antigen recognition is a critical event for T cell proliferation and effector function. Prior studies have identified several transcription factors that contribute to the activity of the IL-2 promoter in stimulated T lymphocytes. Here we describe a novel regulatory element within the IL-2 promoter located immediately upstream of the nuclear factor of activated T cell (NFAT) domain. This region (termed the zinc finger protein binding region (ZIP)) serves as binding site for two differently regulated zinc finger proteins: the constitutively expressed transcription factor Sp1 and the inducible early growth response protein EGR-1. In unstimulated cells which do not secrete IL-2, only Sp1 binds to this region, while in stimulated IL-2 secreting cells the inducible EGR-1 protein recognizes this element. In Jurkat T cells, the ZIP site serves as an activator for IL-2 gene expression, and a combination of ZIP and NFAT binding sites is required for maximal IL-2 promoter activity. These results suggest a critical role of the ZIP site for IL-2 promoter activity.

MEDLINE:95385995

**Activation of the HIV-1 enhancer by the LEF-1 HMG protein on nucleosome-assembled DNA in vitro.**

Lymphoid enhancer-binding factor 1 (LEF-1) is a regulatory high mobility group (HMG) protein that activates the T cell receptor alpha (TCR alpha) enhancer in a context-restricted manner in T cells. In this paper we demonstrate that the distal region of the human immunodeficiency virus-1 (HIV-1) enhancer, which contains DNA-binding sites for LEF-1 and Ets-1, also provides a functional context for activation by LEF-1. First, we show that mutations in the LEF-1-binding site inhibit the activity of multimerized copies of the HIV-1 enhancer in Jurkat T cells, and that LEF-1/GAL4 can activate a GAL4-substituted HIV-1 enhancer 80- to 100-fold in vivo. Second, recombinant LEF-1 is shown to activate HIV-1 transcription on chromatin-assembled DNA in vitro. By using a nucleosome-assembly system derived from Drosophila embryos, we find that the packaging of DNA into chromatin in vitro strongly represses HIV-1 transcription and that repression can be counteracted efficiently by preincubation of the DNA with LEF-1 (or LEF-1 and Ets-1) supplemented with fractions containing the promoter-binding protein, Sp1. Addition of TFE-3, which binds to an E-box motif upstream of the LEF-1 and Ets-1 sites, further augments transcription in this system. Individually or collectively, none of the three enhancer-binding proteins (LEF-1, Ets-1, and TFE-3) could activate transcription in the absence of Sp1. A truncation mutant of LEF-1 (HMG-88), which contains the HMG box but lacks the trans-activation domain, did not activate transcription from nucleosomal DNA, indicating that bending of DNA by the HMG domain is not sufficient to activate transcription in vitro. We conclude that transcription activation by LEF-1 in vitro is a chromatin-dependent process that requires a functional trans-activation domain in addition to the HMG domain.

MEDLINE:95370270

**HIV-1 envelope glycoproteins induce activation of activated protein-1 in CD4+ T cells [published erratum appears in J Biol Chem 1995 Dec 1;270(48):29038]**

Activation of CD4 positive T cells is a primary requirement for human immunodeficiency virus (HIV) entry, efficient HIV replication, and progression to AIDS, Utilizing CD4 positive T cell lines and purified T cells from normal individuals, we have demonstrated that native envelope glycoproteins of HIV, gp 160, can induce activation of transcription factor, activated protein-1 (AP-1). The stimulatory effects of gp160 are mediated through the CD4 molecule, since treatment of gp160 with soluble CD4-IgG abrogates its activity, and CD4 negative T cell lines fail to be stimulated with gp160. Immunoprecipitation of the gp 160-induced nuclear extracts with polyclonal antibodies to Fos and Jun proteins indicates that AP-1 complex is comprised of members of these family of proteins. The gp160-induced AP-1 complex is dependent upon protein tyrosine phosphorylation and is protein synthesis-independent. This stimulation can also be abolished by inhibitors of protein kinase C, but it is unaffected by calcium channel blocker or cyclosporine A. This gp160 treatment adversely affects the functional capabilities of T cells: pre-treatment of CD4+ T cells with gp160 for 4 h at 37 degrees C inhibited anti-CD3-induced interleukin-2 secretion. Effects similar to gp160 were seen with anti-CD4 mAb. The aberrant activation of AP-1 by gp160 in CD4 positive T cells could result in up-regulation of cytokines containing AP-1 sites, e.g. interleukin-3 and granulocyte macrophage colony-stimulating factor, and concurrently lead to T cell unresponsiveness by inhibiting interleukin-2 secretion.

Note:

The yellow section shows that the proteins (marked with red) have the interaction relationship.
